# Attribute-Based Encryption Schemes for Next Generation Wireless IoT Networks: A Comprehensive Survey

**DOI:** 10.3390/s23135921

**Published:** 2023-06-26

**Authors:** Shruti, Shalli Rani, Dipak Kumar Sah, Gabriele Gianini

**Affiliations:** 1 Goswami Ganesh Dutta Sanatan Dharma College, Chandigarh 160030, India; shruti@ggdsd.ac.in; 2 Chitkara University Institute of Engineering and Technology, Chitkara University, Punjab 140401, India; 3 Department of Computer Engineering and Applications, GLA University, Mathura 281406, Uttar Pradesh, India; dipak.sah@gla.ac.in; 4 Dipartimento di Informatica, Università degli Studi di Milano, Via Celoria 18, 20133 Milano, Italy

**Keywords:** attribute-based encryption, cryptographic methods, fine-grained access control, Internet of Things, security, privacy

## Abstract

Most data nowadays are stored in the cloud; therefore, cloud computing and its extension—fog computing—are the most in-demand services at the present time. Cloud and fog computing platforms are largely used by Internet of Things (IoT) applications where various mobile devices, end users, PCs, and smart objects are connected to each other via the internet. IoT applications are common in several application areas, such as healthcare, smart cities, industries, logistics, agriculture, and many more. Due to this, there is an increasing need for new security and privacy techniques, with attribute-based encryption (ABE) being the most effective among them. ABE provides fine-grained access control, enables secure storage of data on unreliable storage, and is flexible enough to be used in different systems. In this paper, we survey ABE schemes, their features, methodologies, benefits/drawbacks, attacks on ABE, and how ABE can be used with IoT and its applications. This survey reviews ABE models suitable for IoT platforms, taking into account the desired features and characteristics. We also discuss various performance indicators used for ABE and how they affect efficiency. Furthermore, some selected schemes are analyzed through simulation to compare their efficiency in terms of different performance indicators. As a result, we find that some schemes simultaneously perform well in one or two performance indicators, whereas none shines in all of them at once. The work will help researchers identify the characteristics of different ABE schemes quickly and recognize whether they are suitable for specific IoT applications. Future work that may be helpful for ABE is also discussed.

## 1. Introduction

The Internet of Things (IoT) has been growing day by day in many application areas in the last few years. IoT can be defined as a network of physical devices that are equipped with sensors and software to communicate and exchange data with other systems and devices over the internet. These can include everything from common household items to complex industrial machines. Numerous services and the rapid evolution of IoT are entering different fields such as healthcare, farming, industries, research work, and many more. Some IoT applications include: WSN (wireless sensor networks) [1], smart city [2], home automation [3], logistics management, smart cars [4,5], smart grid [6], retail management, and healthcare. One of the most common and recent uses of this technology can be illustrated with an example from healthcare: the medical details of the remote patient can be collected from the sensors attached to the patient’s body and transmitted to the server, where doctors and nurses can access it according to their provided privileges. In this way, patients can be monitored regularly and diagnosed without the need to visit them physically.

The volume of data collected by IoT devices is generally huge; data are often stored on servers that can be trusted or untrusted (for example, managed by some third party). Moreover, data can be public or private depending on the type of information being carried; protecting them from unauthorized access by any outside body is important (an unauthorized user could collect, decipher, and misuse the data). Typically, data on servers are saved in an unreadable form, but the classic approaches used to protect communication are not sufficient to protect it against internal threats and system vulnerabilities [7,8]. Therefore, encryption-based systems are needed. Any encryption scheme should fulfill the following requirements:

1. Security: An ideal security mechanism should have the ability to protect against various attacks and keep user data safe in any situation. The more security measures are added, the more difficult it becomes to attack the data. Security ensures integrity and prevents loss or corruption of data. Some of the measures that can be taken are:

- Firewall;
- Improved IDS (Intrusion Detection System);
- Using the cloud to store data in an encrypted manner;
- Setting up data centers that are physically secure.

2. Access Control: This limits access to the data and is controlled by the data owner. You can decide which clients have permission to view specific information while denying it to others using authentication and authorization techniques. In this way, data can be protected from unauthorized users. It requires:

- Configuring access rights instantly;
- Data emergency lockdown;
- Easily creating and administering access control groups.

3. Privacy: Similar to security and access control, privacy is equally important. It refers to protection of personal or sensitive information transmitted/stored over the network, ensuring that any unauthorized individual is not able to access, view, or change data without proper authorization. It can be best provided by:

- Control over data access;
- Data leak-proof system;
- Avoiding data loss from the database.

Data owners and businesses have access to limitless cloud storage and data outsourcing services, which provide them the ability to store and handle enormous amounts of data, but along with this arises concern about the security and privacy of data. Numerous approaches exist that enable searching for outsourced data that are stored in the cloud to protect data confidentiality, but that are unable to enforce access controls or limit access to a particular data record. Therefore, maintaining data confidentiality, choosing when to access data, and enforcing access controls are major issues in an untrusted environment. To provide secure and effective access control, researchers have identified a variety of system models based on cryptographic processes. Although these methods make it possible for a large number of people to share data, there are still certain unresolved problems, especially concerning applications of the Internet of Things. First, access control policies should include significant information about individuals, objects, or an environment exposed to constantly changing circumstances and connected to ongoing actions in the specific setting. Second, since IoT applications vary greatly, the security of these applications using a collusion-resistant system and offering fine-grained access control is a major task. The vulnerability of application data can be harmful to systems and users. Thirdly, it is important to understand how a user joins/leaves the system with minimum computing overhead. A significant amount of research has been done to create the cryptography approaches necessary to address these aforementioned problems.

The traditional symmetric cryptography approach uses the same key for both encryption and decryption, which encounters key distribution and management issues in large distributed settings like the cloud. The traditional asymmetric cryptography method is computationally inefficient, as encryption is done using a public key and decryption using a private key. Various cryptographic access control methods have been proposed to reduce the computing cost associated with standard cryptographic operations and meet objectives, but none of them has been found to be efficient and provide access control better than ABE (attribute-based encryption).

One of the promising encryption models for the Internet of Things is now assumed to be ABE. It was introduced in 2005 by Sahai and Waters [9], to provide fine-grained access control to encrypted data. The authenticity of the user is identified using the ciphertext and a key that depends on a certain set of attributes; then, only data can be decrypted. This type of technology uses elliptic curve cryptography and has two paradigms: KP-ABE (key policy attribute-based encryption) and CP-ABE (ciphertext policy attribute-based encryption). Some of the aspects that need to be evaluated to qualify for an ideal ABE scheme are discussed below [10]:

1. Accountability of the user: If an authorized user engages in unethical behavior by sharing their private key with an unauthorized user, the user’s accountability is questioned, and the problem must be resolved.

2. Revocation of the user: Revokes the user’s right when he exits the system. Revocation ensures that other users who share the same space with others are secure. This can be done by:

- Determining whether or not a user is active;
- User rights are revoked by the owner accordingly.

3. Scalability: This defines how flexible the system is so that the owner can provide services according to the demand of the system. Any change in the requirements of the services or resources can be changed instantly by the owner.

4. Confidentiality of data: Confidentiality means that only authorized users can access the encrypted data.

5. Unaffected by collusion: The ability to withstand an attack by combining numerous attributes in key generation to decrypt the ciphertext related to the targeted person. This type of attack is initiated by unauthorized users or attackers using various attributes obtained by hacking or with the help of users.

6. Fine-grained access: This is also one of the distinguishing characteristics of the services provided by cloud computing. Maintaining data access rights grants new user access controls and total control over how and to whom data are disclosed.

**Figure 1:**
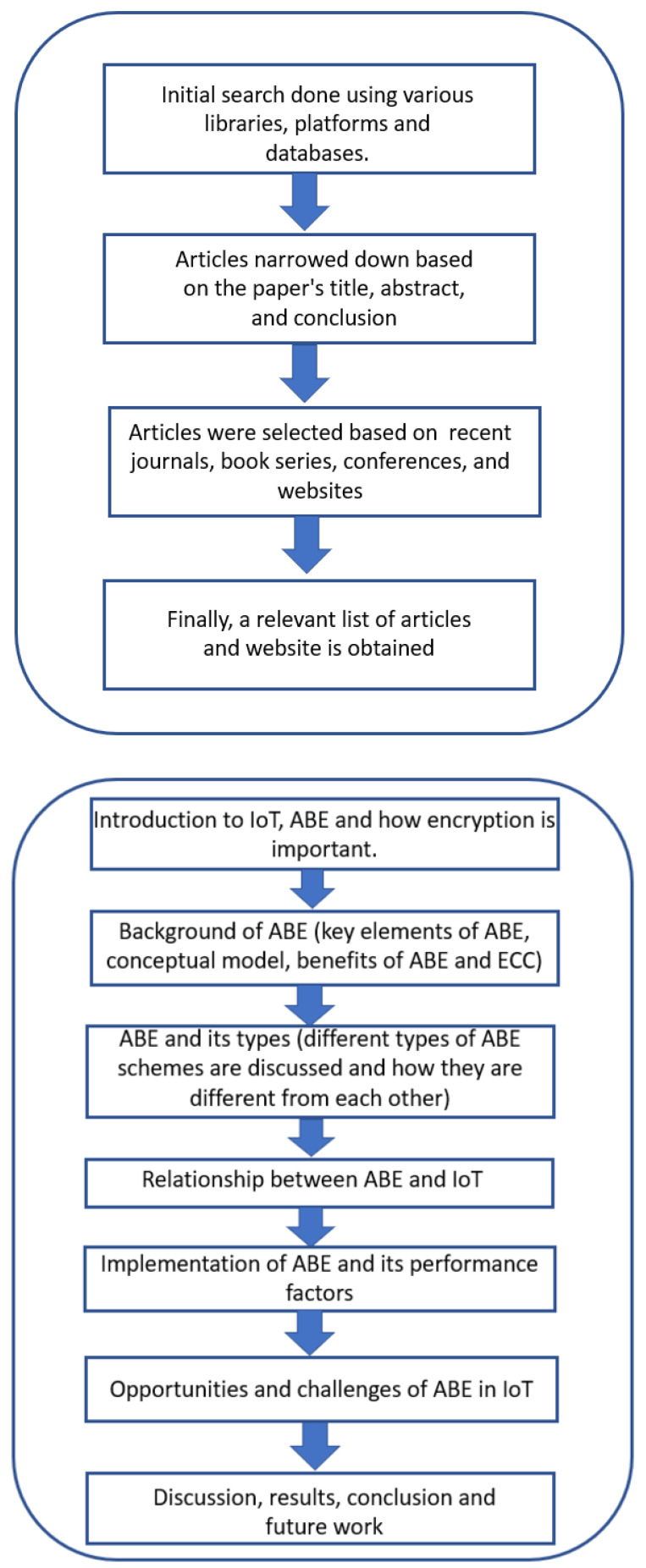
Screening and review methodology of the paper.

*Survey method:* To identify issues related to encryption, security, and privacy using ABE and its various aspects, a review was performed by selecting relevant articles through a screening process. The search for articles was conducted using keywords such as “Attribute-based Encryption”, “Fine-grained access”, and ”Internet of Things” in various databases, including Google Scholar, Research Gate, Web of Science, and Scopus. Initially, many articles were selected. In the second search, articles were narrowed down according to title, abstract, and conclusion. Finally, articles were selected based on recent journals, book series, conferences, and websites. The screening process and review methodologies are highlighted in a schematic diagram presented in Figure 1. Research papers were selected on the basis of the ABE scheme even if they were not suitable for IoT applications, but there are still a few exceptions [11]. Some of the papers were excluded even after ABE was mentioned in them due to (i) the scheme was obsolete compared to others, and (ii) there was no security proof. In this way, research papers were shortlisted for further analysis in this survey. The authors meticulously analyzed and discussed the collected information related to ABE and IoT, as well as future research work from the selected articles.

*Motivation:* The motivation behind using ABE in IoT is to provide a secure and flexible data access control mechanism. With ABE, data can be encrypted with attributes instead of traditional cryptographic keys, and access to the data can be granted based on a set of attributes owned by the requestor. This is helpful in IoT scenarios where a large number of devices are connected to the network, each having a different set of capabilities and access levels.

This survey helps us identify advanced research and developments and allows researchers to understand existing techniques, challenges, and opportunities associated with this area. Researchers can also gain insight into the different approaches to implementing ABE in IoT, the strengths and weaknesses of these approaches, and the research gaps that need to be addressed. It also allows researchers to identify key application areas where ABE can be applied to address security and privacy concerns. Overall, a foundation is built to develop new solutions and approaches that can improve security and privacy using ABE.

*Contribution:* This survey reviews ABE models suitable for IoT platforms, keeping in mind the desired characteristics. There are three main key performance indicators known as KPIs—namely, CPU efficacy of the data generator, bandwidth effectiveness of the data generator, and bandwidth effectiveness of the authority key—that are considered when choosing the appropriate ABE scheme for a specific application in IoT. There are also three accessory performance indicators known as APIs that are of less importance than KPIs in IoT, namely, the storage ability of the data generator, CPU efficacy of the data user, and bandwidth effectiveness of the data user. This survey considers only those ABE schemes that have one or more KPIs/APIs that are implemented in IoT networks and that have security proofs. It will help researchers identify characteristics of different ABE schemes quickly and recognize whether it is suitable for a specific IoT application or not. There are a good number of ABE surveys [12,13,14,15,16,17,18,19,20]; some surveys are outdated as new and improved ones are proposed, some articles show very limited contributions, and some focus only on specific characteristics. We also simulate different schemes to evaluate their efficiency, as a result of which we find that the schemes excel in one or two performance indicators only instead of all three. The outcomes of our review are summarized below:

- Background of attribute-based encryption (ABE) scheme;
- The key elements, conceptual model, and types of ABE schemes are discussed;
- Relationship between ABE and IoT and how ABE can be implemented in IoT applications;
- Various performance indicators of ABE are discussed, as well as how their performance can be improved and implemented in specific IoT applications;
- Opportunities and challenges faced by ABE in the IoT environment.

The paper is organized into ten sections. Section 1 is an introduction that discusses IoT, encryption, how encryption is important for data safety, ABE, and how the papers were selected for the survey. Section 2 is about the background of the ABE scheme, its key elements, the conceptual model, and the benefits. Section 3 explains various types of ABE schemes, their advantages and disadvantages, and a comparison among these types is also made. Section 4 is about security schemes other than ABE that have been used in various domains. Section 5 shows the relationship between ABE and IoT, and the architecture of the IoT network with ABE is mentioned. In Section 6, the implementation of the ABE scheme as KP-ABE and CP-ABE is discussed. Various performance factors of both variants are analyzed, and factors required to improve them are studied. In the same section, various ABE schemes, their type, and in which universe they fall are shown in tabular form. Section 7 is about the opportunities and challenges of ABE in the IoT environment. An evaluation of ABE schemes on the basis of their performance factors is made in Section 8. Section 9 shows the result of various KP-ABE and CP-ABE schemes compared on the basis of key performance factors in graphical form. Finally, Section 10 is about future work that can be completed in the field of ABE, thus concluding the paper. Figure 1 shows the screening process and review methodology used in this document.

## 2. Background for Attribute-Based Encryption

Attribute-based encryption (ABE) is a cryptographic approach that allows fine-grained access control to encrypted data. Unlike conventional encryption techniques that require a secret key to decrypt data that only authorized users have access to, ABE enables data to be encrypted with attributes like a user’s role or location. Access to the data is given depending on the attributes of the person requesting it. In 2005, Sahai and Waters [9] introduced the notion of ABE as a framework for using attributes to manage encrypted data access. Since then, ABE has drawn a lot of attention from researchers and has been used in various fields, such as cloud computing, wireless networks, and the IoT.

ABE presents several advantages over traditional encryption methods. It allows for fine-grained access control and enables the granting or revocation of data access based on dynamic and flexible circumstances, such as changes in user location or role. This feature makes ABE particularly suitable for IoT environments where numerous devices with various capabilities and access levels are connected to the network. Since only users whose attributes satisfy the access policy can decrypt the data, ABE also offers data confidentiality and privacy. Accordingly, even if an attacker obtains access to the encrypted data, they will not be able to decrypt it unless they have the required attributes. However, ABE has some limitations as well. For example, the decryption process can be computationally intensive because it requires the evaluation of access policies. Moreover, the usage of ABE could make ciphertexts larger, which can make it difficult to store and transmit large volumes over the network. A comparative analysis between the traditional cryptography scheme and the ABE scheme is shown in Table 1.

### 2.1. Key Elements of ABE

The key elements of attribute-based encryption (ABE) are as follows:

1. Attributes: A set of attributes is used to define the access control policy. Attributes can represent various characteristics such as user identity, role, location, or any other relevant information.

2. Access policy: This defines the rules for accessing encrypted data based on the attributes of the user or the requester. The access policy can be expressed as Boolean expressions or logical predicates.

3. Encryption: Encryption is a process of encrypting data using the access policy and a public key.

4. Decryption: Decryption is a process that involves evaluating the access policy against the attributes provided by the requester. If the attributes match the access policy, the data are decrypted using a private key.

5. Key management: This is required to generate and distribute public and private keys to authorized users. The key management system is responsible for managing access control policies, attributes, and keys.

### 2.2. ABE Conceptual Model

The conceptual model of ABE aims to enable fine-grained access control over encrypted data using attributes to define access policies. The model is composed of various entities that collaborate to ensure that only authorized users can have access to the data. The components present in the model are:

1. Attribute authority: This is responsible for assigning attributes to users based on their identity, role, or any other related information. Attribute authority also defines the access control policy based on attributes.

2. Key authority: This plays an important role in generating and distributing public and private keys to authorized users. The key authority is also responsible for implementing an access control policy by controlling the issuing of private keys based on the attributes provided by the user.

3. Data owner: A data owner is responsible for encrypting the data using the access control policy and a public key. He can specify the attributes required to access the encrypted data.

4. User: One who requests access to the encrypted data is a user. The user has a set of attributes that are evaluated against the access control policy. If the attributes satisfy the policy, the user can decrypt the data using the private key issued by the key authority.

### 2.3. Benefits and Drawbacks of ABE

The implementation of the ABE scheme instead of traditional encryption methods has several benefits, some of which are discussed below:

1. Fine-grained access control: ABE allows access to data based on the attributes of the user. This makes it more dynamic and adaptable to access control policies, particularly for the IoT, where a lot of devices with various capabilities and access levels are connected.

2. Improved data confidentiality and privacy: ABE ensures privacy and confidentiality of data by allowing only authorized users to access the data, as it can only be decrypted by users whose attributes match the access policy defined by the data owner.

3. Reduced data exposure: ABE limits data exposure by only allowing users with the appropriate attributes to access the data. As a result, the chances of unauthorized access and data breaches are reduced.

4. Scalability: ABE is capable of managing a significant number of users and attributes that make it suitable for systems such as wireless networks, cloud computing, and IoT.

5. Simplified key management: ABE eliminates the need for separate keys for each user, reducing the risk of key exposure or loss.

6. Revocation: ABE enables revocation of access by revocation of user attributes. Because of this, access can be revoked without having to change the encryption key, which is useful when access control restrictions are changed frequently.

7. Dynamic access control policies: ABE allows for dynamic access control policies that can be updated or revoked as needed. This enables more fine-grained and flexible control over data access, which is useful in IoT.

8. Access delegation: ABE allows for delegation of access rights, enabling a user to grant access to others without having to share the decryption key; this is useful in situations where a user needs to share access to data with others temporarily.

9. Flexibility: ABE can be deployed in various settings such as cloud computing, fog computing, wireless networks, and mobile systems.

10. Anonymity: ABE enables data encryption in such a way that when data are decrypted, the identity of the user is preserved. It is helpful in crucial circumstances.

While attribute-based encryption (ABE) methods have several benefits, they also have some drawbacks. These include difficulties in management and execution, especially when designing and managing access control policies for attributes. In decentralized systems with several attributes and frequent updates, key management can become difficult. Additionally, in comparison to traditional encryption schemes, ABE adds more computational overhead, which can affect performance. Security issues may also arise from trust assumptions. Therefore, before using ABE, a careful assessment and consideration of these drawbacks is required, along with the appropriate measures and design. Table 2 shows the benefits and drawbacks of ABE.

### 2.4. Elliptic Curve Cryptography

Elliptic curve cryptography (ECC) is a key-based technique used to decrypt and encrypt data. It is a public key encryption method that uses elliptic curve theory to produce cryptographic keys that are quick, smaller, and more effective. ECC combines two keys through a mathematical procedure that is then used to encrypt and decrypt data. One of them is a private key that is only known by the sender and recipient of the data, while the other is a public key that is known to everyone.

ECC has been accepted for public key encryption in various application areas (mainly where sensors are used), due to its small key size and relatively quick computations (for example, while RSA requires a 1024-bit key to achieve an 80-bit security level, ECC only requires a 160-bit curve) [21]. ECC is similar to other public key encryption techniques like RSA and Diffie–Hellman.

The idea of a one-way function is used in these cryptographic techniques. This implies that getting from point A to B is simply a mathematical equation using public and private keys. However, without knowing the private key and size of the key, traveling from point B to point A is challenging.

### 2.5. Attacks in ABE

Fault attacks and side-channel attacks (SCA) are two attacks that are threats to ABE. Fault attacks exploit vulnerabilities in the implementation of the cryptographic algorithm by introducing intentional faults. However, SCA exploits information leaked during the execution of the algorithm, such as power consumption or timing variations [22,23,24]. In relation to ABE, fault attacks can be used as a form of SCA, allowing attackers to gain unauthorized access to encrypted data. Countermeasures such as fault detection and correction, secure hardware implementation, and side-channel analysis-resistant techniques can help reduce these risks and ensure the security of ABE systems. Power analysis attacks take advantage of patterns in power usage to collect private data. These methods seek to minimize power variations and prevent the disclosure of sensitive data when performing cryptographic procedures. ABE countermeasures for power analysis attacks include methods to reduce power variations and protect sensitive data. Some countermeasures include: (1) Power analysis resistance: ABE schemes with implementations resistant to power analysis can make it more difficult for attackers to gather relevant data from power variations. These designs might make use of randomization, masking, or power consumption balance. (2) Side-channel analysis-resistant implementations: It is vital to create ABE implementations that can withstand side-channel attacks, such as power analysis attacks. It is possible to eliminate power variances that attackers could use as an advantage by using methods such as blindness, masking, or constant-time algorithms. (3) Hardware countermeasures: This includes using hardware-based approaches, such as randomizing power use, using components resistant to DPA, or putting in place secure hardware modules. (4) Secure implementation practices: Maintaining secure coding standards and practices, such as constant-time implementations, data-independent operations, and appropriate key management, to reduce data leakage detected by power analysis.

Differential power analysis (DPA) and differential fault analysis (DFA) [25,26] are attacks that target cryptographic systems by analyzing power consumption and injecting faults, respectively. These attacks can threaten ABE schemes by exploiting vulnerabilities in the underlying cryptographic operations. To thwart these attacks in ABE, combined measures can be used, such as protected implementation techniques, error detection and correction mechanisms, side-channel analysis resistance, and physical security measures. These measures aim to reduce power variations, detect and correct injected faults, resist side-channel analysis, and protect hardware implementation. The effectiveness of these countermeasures depends on the specific ABE scheme and its implementation, which requires thorough security analysis and testing.

## 3. ABE and Its Types

ABE was constructed for improved security and access control [27]. It is an advanced cryptographic method that removes the shortcomings of old public key cryptography and has no restriction on the number of users. These factors have been taken into account when developing this method, as it uses attributes as an access policy to control users’ access to cloud data. The entities used for the implementation of ABE are the sender, who owns the data, the receiver, who uses the data, and a key generation authority for the encryption and decryption of data. Some policies known as access policies (APs) are also created, which are responsible for the decryption of data at the receiver end, where only the authorized user with the required attributes can decrypt the ciphertext.

A data owner uses ABE and a public key for key encryption after data encryption, making use of a symmetric encryption method and a symmetric key. A group of users receives the encrypted key as a ciphertext. The private key is provided to all users for the decryption of the encrypted key. In this instance, the data owner does not need to be aware of the identities and dynamic behavior of the authorized users. Some of the requirements to be fulfilled by any ABE scheme are discussed below.

1. Confidentiality and privacy of the data restrict the information to protect it. Both of these are important for cloud storage, as the data held by the service provider are vulnerable to attack. Access to the data should only be available to specific authorized people. Using ABE allows data access in the absence of an encrypter and protects the privacy of the data user.

2. According to the credentials provided by the connected systems, fine-grained access allows various privileges to users even if they are in the same group and is flexible in specifying access rights to individual users.

3. In order to implement fine-grained access control, it is crucial that the APs given by the data owner be expressive. ABE is also necessary to support the policy expressiveness. The access control resembles the real-world access control because of this necessity.

4. The increase in system users should not have an impact on the system’s performance.

5. To meet all requirements with the least amount of calculation cost, the computation overhead is crucial.

6. The system must stop collusion attacks that merge their data in order to obtain unauthorized data illegally by collaborating. In the cloud, such attacks can either revoke users who attempt to obtain the original material or might be a collection of misbehaving system users working together to combine their information and obtain higher access permissions.

7. The related access control strategy must be able to limit or revoke a user’s access rights when they are degraded or depart the system, respectively, without incurring a large computational expense. ABE’s attribute update method is also complicated and difficult to handle because changing just one attribute could have an adverse effect on several users that access the same attribute.

**Figure 2:**
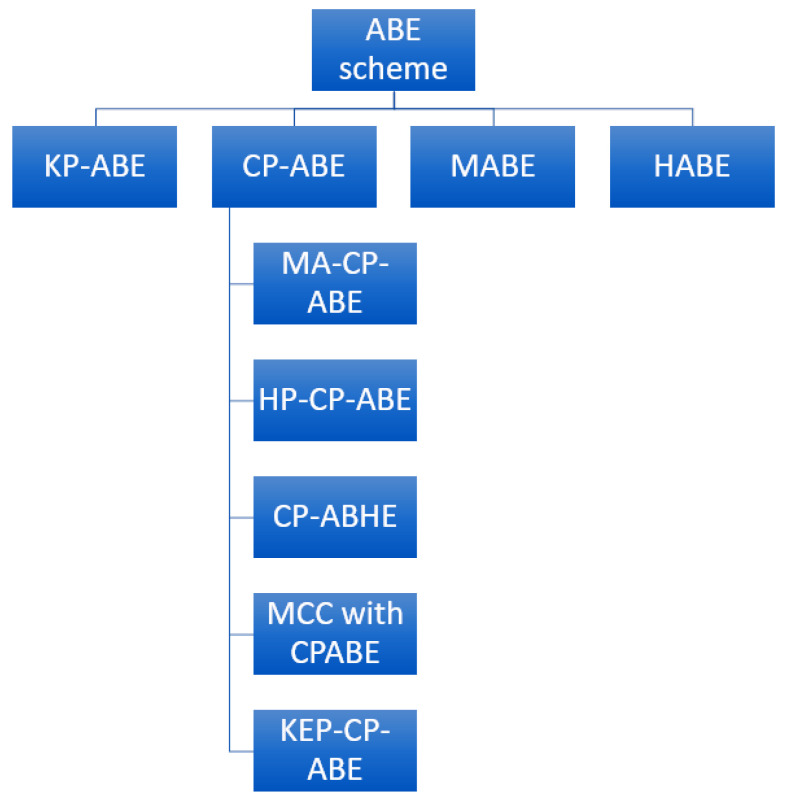
Different types of ABE schemes.

The ABE encryption scheme has different types (Figure 2) that we study in this section and review in the survey. All the advantages and disadvantages of various schemes are also discussed.

1. KP-ABE (key policy attribute-based encryption scheme): Here, AP is used to create ciphertext, and user attributes are used for decryption. Every private key has an access structure that only permits a specific ciphertext to be decrypted by that key. First, a public key (PK) and a master key (MK) are generated, after which a random value is selected from the attribute set that creates a ciphertext of the data, thus completing the encryption process. Only if the user’s attribute fits the AP is a key generated, allowing encryption [28]. Some of the disadvantages of KP-ABE are:

- Several parties cannot perform cooperatively;
- As the sender must be dependent on the key allocator, ABE insecurely allows control over decryption.

2. CP-ABE (ciphertext policy attribute-based encryption schemes): In CP-ABE, for encryption, AP is created using user attributes, and the receiver can decrypt the data only if their attributes are satisfied by the conditions of AP. Its functioning is opposite that of KP-ABE. Firstly, PK and MK are generated; then, using AP and PK, a ciphertext is created. The secret key is generated using MK and an attribute set. One of the disadvantages of CP-ABE is that it is less resistant to collusion attacks. During the study, we found that CP-ABE also has the types described below.

- MA-CP-ABE (multi-authority ciphertext attribute-based encryption scheme) [29]: This is implemented on vehicular data, creating a totally practical environment by merging the multi-authority factor with the original CP-ABE.
- HP-CP-ABE (hidden policy ciphertext attribute-based encryption scheme) [30]: This can handle big data problems in cloud services. HP-CP-ABE employs a hidden policy with CP-ABE to address problems of data leakage and increased computing costs.
- CP-ABHE (ciphertext policy attribute-based hierarchical document collection encryption scheme) [31]: This includes a hierarchical concept and claims to fulfill enterprise requirements, overcoming the issue of low efficiency when dealing with large files. The access structure associated with ciphertext in CP-ABHE is hierarchical tree-based. It also claims to reduce the size of ciphertext while maintaining its security and increasing efficiency dramatically.
- MCC with CP-ABE (mobile cloud computing with CP-ABE) [32]: This was created while working for a secure and lightweight fine-grained data sharing scheme (SLFG-DSS), which is an access structure for cloud computing. This scheme involves the creation of a secret key that changes while returning from the server. ABE is stated to be unsuitable for MCC because it is too expensive, even with the adopted strategy, so the MCC CP-ABE was proposed. The scheme proposes that the heavy data computed with the restricted mobile resources be sent to a remote server for storage.
- KEP-CP-ABE (key exchanging policy with CP-ABE) [33]: This focuses on making the fog computing environment more secure using the ABE scheme using the concept of CP-ABE. KEP-CP-ABE claims to give positive results without compromising efficiency.

3. MABE (multi-authority attribute-based encryption schemes) [34]: There are several different attribute authorities in this scheme that generates and handles secret keys. To decrypt a message, the user needs to have a set of all authority in the system. MABE suffers from a disadvantage in that each authority set needs to be different or, we can say, disjoint.

4. HABE (hierarchical attribute-based encryption scheme) [35]: A user is provided with a hierarchy of domain authority and attributes where the secret key (SK) is a leaf. In this hierarchical structure, the domain authority present in the leaf cannot decipher the ciphertext associated with its root. The key generation process determines the level of authority and may generate the SK for the future. The user’s attributes are stored in the SKs for that particular user. The access structure within the ciphertext C was used by a random algorithm, and its decryption involves the user’s key to fulfill the access structure. The scheme has a disadvantage in that the root authority is able to decrypt all ciphertext below it, but if it gets compromised, everything will be at risk. Moreover, HABE implementation results in a high computational overhead.

A comparison between ABE and some of its types is made in Table 3 below.

The above ABE schemes are the ones most used in various domains. However, there are also some recent and lesser-known schemes, which are discussed below:

1. ABBE (Attribute-Based Broadcast Encryption): ABBE is designed to broadcast messages to a subset of users based on their attributes. It allows the broadcaster to encrypt a message under an access policy that specifies the attributes that are required to decrypt the message.

2. F-ABE (Fuzzy ABE): This type of ABE introduces the concept of fuzzy attributes, where attributes can have degrees of membership. It allows for more flexible access control based on fuzzy matching of attributes.

3. FG-ABE (Fine-Grained ABE): FG-ABE aims to provide even finer-grained access control by implementing additional context or conditions into access policies. It allows for access control based on dynamic attributes, time-based constraints, location, or other factors.

4. ABS (Attribution-Based Signatures): ABS is a cryptographic scheme that combines the concepts of ABE and digital signatures. It allows users to sign messages using their attributes as credentials, and the verification process is performed according to the access policy associated with the signature. ABS enables secure and selective disclosure of attributes in digital signature schemes.

5. O-ABE (outsourced ABE): This focuses on securely outsourcing the encryption and decryption operations to a third party, such as cloud service providers. It ensures that sensitive data remain protected even when cryptographic operations are performed by untrusted parties.

6. ABFE (attribute-based functional encryption): This is a variant of ABE that focuses on encrypting data based on functions rather than attributes. It allows users to compute specific functions on the encrypted data without revealing the underlying plaintext. ABFE enables secure computation and sharing of data while preserving privacy.

7. ABRE (attribute-based revocable encryption): ABRE extends ABE by incorporating mechanisms for attribute revocation. It allows efficient revocation of users or attributes, ensuring that revoked users or attributes cannot decrypt new ciphertexts while maintaining the security and functionality of the system.

## 4. Related Work in ABE and Other Security Schemes

IoT being the foundation for smart villages helps to enable real-time data analysis and automation that benefit the villagers in various fields such as agriculture, healthcare, transport, etc. However, IoT devices face security challenges when using public networks, and cloud-based processing causes network congestion. Integrating distributed fog computing (DFC) with IoT improves security and privacy for villagers and consumer electronic (CE) devices. Ref. [36] explores DFC-IoT integration, presents a case study on an intrusion detection system in a DFC-based smart village, and discusses open security issues in fog-to-things-enabled smart villages. The recent development in wireless communication systems and IoT has led to the development of zero-touch networks (ZTNs). These provide self-configuring and automated service-level policies. As insecure data exchange over public channels carries security risks, Ref. [37] analyzes attack surfaces and architectural flaws in ZTNs enabled by IoT. It presents a deep learning and blockchain-assisted case study for secure data sharing, including a novel intrusion detection system and an authentication protocol. The experimental results validate the approach. The article highlights critical issues, opportunities, and open research directions in this domain. Business intelligence (BI) involves the use of tools to gain insight and make informed decisions. Integrating IoT data with BI systems poses security and privacy challenges due to data inference and poisoning attacks. The study [38] proposes an integrated architecture to improve security and privacy in BI applications based on IoT. It includes an intrusion detection engine and a two-level privacy engine. The results and analysis show that the performance of the scheme is superior compared to existing privacy-preserving algorithms.

Fault detection of Ring-LWE (learning with errors) fault detection on FPGA (field-programmable gate arrays) is the process of designing and implementing systems to identify and mitigate faults or errors that can arise during the execution of cryptographic algorithms based on the Ring-LWE problem. While implementing Ring-LWE on an FPGA, a fault can occur for a number of reasons, such as hardware issues, external variables, or malicious attacks that can affect the correctness and security of the cryptographic computations. To solve this problem, fault detection techniques such as redundancy, error-checking codes, parity bits, or error-detection algorithms can be used to find and fix errors that occur while Ring-LWE algorithms are running on an FPGA. They can also send alerts for more in-depth investigation. The purpose of fault detection is to guarantee the accuracy and dependability of the Ring-LWE cryptographic calculations on FPGA even in the presence of errors or faults [39,40].

NIST lightweight standardization focuses on developing cryptographic standards for resource-constrained devices like IoT. It aims to provide secure and efficient algorithms tailored to limited computational power and memory. The process involves evaluating proposed algorithms and selecting standards to ensure interoperability and security in lightweight applications. It promotes the widespread adoption of secure IoT and embedded systems. Fault detection techniques are applied to the Pomaranch cipher [41,42,43] to identify and detect faults or errors in the architecture. These techniques involve analyzing the internal structure and operations of the cipher and detecting any abnormalities in the expected behavior. The goal of implementing fault detection mechanisms is to ensure the reliability and correctness of the cipher’s operations, thereby preventing potential vulnerabilities or attacks. Another is the Grostl hash architecture [44], whose purpose is to design and implement reliable architectures for the Grostl hash function. It enhances the resilience and dependability of the hash function by incorporating fault-tolerant mechanisms and ensures that the hash function can operate reliably even in the presence of faults. At the same time, it maintains the integrity and security of hash computations. The diagnosis of low-energy Midori cipher faults [45,46] is another technique to diagnose and identify faults or vulnerabilities in a low-energy Midori cipher. By analyzing the behavior and output of the cipher, it can detect inconsistencies that may indicate the presence of faults. In addition, fault diagnosis helps to understand potential weaknesses in the cipher and allows appropriate steps to be taken. In the case of the RECTANGLE cipher, a fault diagnosis technique can be used. Any change in behavior can lead to the detection of potential faults. The diagnosis of faults helps uncover weaknesses in the cipher and allows for the development of suitable mitigation strategies [47,48,49].

Post-quantum cryptography (PQC) [50,51] refers to cryptographic techniques designed to resist attacks from quantum computers. It is a method based on different mathematical problems that are believed to be difficult for quantum computers. The goal is to ensure the security of data and communication in the presence of powerful quantum computers. PQC involves developing new algorithms, promoting their adoption, and ensuring compatibility with existing systems. Its importance grows as quantum computers advance, protecting our digital infrastructure and sensitive information from future quantum attacks. There are many other cryptography schemes that are also discussed here. Curve448 and Ed448 [52,53,54] are elliptic curve cryptography schemes that provide secure key exchange and digital signatures. They are implemented on the Cortex-M4 microcontroller, which is a low-power embedded processor. This implementation aims to provide efficient and secure cryptographic operations on resource-constrained devices such as the Cortex-M4. On the other hand, SIKE (supersingular isogeny key encapsulation) can also be implemented on Cortex-M4 [55,56]. It is a post-quantum key exchange scheme based on isogeny-based cryptography. It enables secure key-exchange operations on constrained devices and focuses on efficiency and security in the face of potential quantum attacks. There is a variant of SIKE, known as SIKE Round 3 [57,58], which refers to the third round of the SIKE standardization process. Its aim is to select the most secure and efficient implementations of SIKE for postquantum security. In this case, SIKE Round 3 is implemented specifically on the ARM Cortex-M4 microcontroller, providing improved security and performance compared to earlier versions. Kyber [59] is another postquantum key encapsulation mechanism based on the LWE problem. It is implemented on the 64-bit ARM Cortex-A microarchitecture, which is a more powerful processor compared to the Cortex-M4. This implementation aims to provide efficient and secure postquantum cryptographic operations on higher-performance devices such as Cortex-A.

Some of the recent work accomplished in ABE is also discussed here (Table 4). In [60], the authors present an enhancement to a lightweight KP-ABE scheme designed for IoT. They identify security vulnerabilities in the original KP-ABE scheme and propose an efficient fix to address them. The enhanced scheme is extended to a hierarchical KP-ABE (H-KP-ABE) scheme, allowing for role delegation in IoT applications. An example is provided to demonstrate the benefits of the delegation feature in an IoT-based healthcare system. The need for a secure and flexible access control encryption scheme for the large amount of sensitive data generated and transmitted by IoT devices is presented in [61]. The authors propose a modified CP-ABE scheme that achieves full security while reducing decryption overhead and ciphertext length. The scheme is designed to be suitable for IoT devices with limited memory and computing capabilities. The feasibility of the proposed scheme is demonstrated through analysis and experiments. In [62], a novel CP-ABE scheme is proposed to address the issue of exposure to sensitive information in plaintext access policies. The scheme incorporates revocation, white-box traceability, and a hidden policy to improve security and privacy. Here, the ciphertext consists of two parts: one encrypts the access policy using attribute values, revealing only attribute names, and the other part contains updated revocation information for user tracing. The proposed scheme is proven to be efficient, secure against chosen plaintext attacks, and able to achieve the desired functionality. Sowjanya et al. [63] propose a secure framework for wireless body area networks (WBANs) in IoT-based healthcare systems. The framework utilizes elliptic curve cryptography-based CP-ABE without bilinear pairing operations to ensure data security. The scheme is secured under the elliptic curve decisional Diffie–Hellman assumption and incorporates user/attribute revocation. In [64], a fully distributed revocable ciphertext policy hierarchical attribute-based encryption scheme (FDR-CP-HABE) is proposed to address scalability and flexibility issues in ABE used in cloud storage. The scheme offers high flexibility and scalability in key delegation and user revocation, while ensuring efficient and lightweight computation during the decryption phase. By outsourcing computation, most of the operations are performed by the cloud server, reducing the computational burden on users. Moreover, the scheme also reduces the storage costs for users compared to similar schemes. The security of the scheme is proven on the basis of the hardness assumption of the decisional bilinear Diffie–Hellman (DBDH) problem.

The work done by the authors in [65] focuses on improving the flexibility, efficiency, and security of attribute-based dual policy encryption (DP-ABE) for cloud deployments. DP-ABE enables two access control mechanisms for encrypted data based on subjective and objective attributes. The proposed scheme introduces two flexible features, encryption and key generation in single-policy modes, along with securely outsourcing key generation, encryption, and decryption operations to cloud servers. This approach minimizes the overhead for PKG, data owners, and users. In [66], registered ABE was introduced that allows users to generate their own secret keys and register associated public keys with a key curator. The key curator aggregates the public keys into a compact master public key. Users can decrypt by combining their own secret keys with helper decryption keys obtained from the key curator. The scheme ensures polylogarithmic sizes for the aggregated public key, helper decryption keys, ciphertexts, and encryption/decryption times. The key curator maintains no secrets, and the scheme generalizes registration-based encryption (RBE) while making black-box use of cryptography. SHARE-ABE is a collaborative approach that enhances ABE for efficient and privacy-preserving data sharing in resource-constrained environments such as IoT devices [67]. It uses fog computing to assign high computational decryption tasks to fog nodes. The approach employs a chained architecture and false attributes to maintain the privacy of the access policy. It also introduces a collaboration attribute that enables users within a group to combine their attributes while satisfying the access policy.

The paper [68] introduces a novel secure video retrieval scheme for smart grids, addressing security threats associated with the storage of sensitive data on semi-trusted cloud servers. The scheme utilizes symmetric searchable encryption with ABE and multi-feature fusion to achieve fine-grained access control and eliminate unauthorized access. By converting videos into keyframe sets and leveraging image secure retrieval techniques, the scheme reduces retrieval overhead and improves accuracy. The security and privacy issues in smart health are addressed in [69] by proposing a CP-ABE solution called PHCA (policy hiding and cloud auditing), ensuring privacy and security while also providing constant decryption costs. The solution includes an effective third-party auditor to ensure data integrity and implements secure outsourcing decryption algorithms that reduce decryption costs for users.

## 5. ABE and IoT

**Figure 3:**
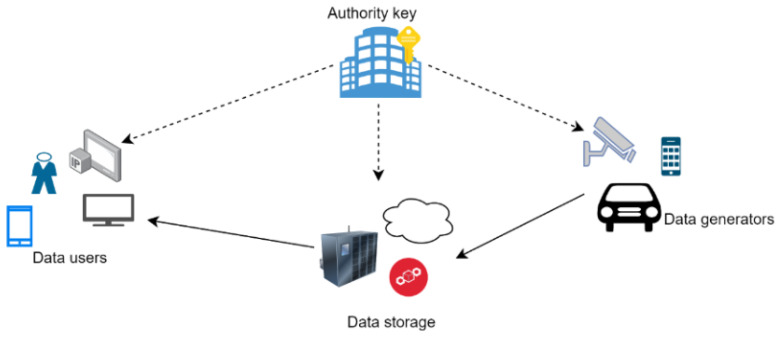
An architecture of the IoT network.

An IoT network architecture (Figure 3) includes producers (data generators), consumers (users), and data storage spaces. Producers are those from where data are produced, and they contain sensors that have some constraints and are battery-powered; consumers are mostly the end users or actuators; and data storage space can be some data centers or the cloud. Storage can be temporary, permanent, or a combination of both. Factors like latency, reliability, scalability, security, energy efficiency, cost, and availability are considered performance metrics in IoT applications. However, for ABE, metrics like encryption and decryption time, key generation authority, communication overhead, computational overhead, storage overhead, scalability, and efficiency of revocation are considered for performance evaluation. A more meaningful relationship between the performance factors ABE and IoT is represented using the following equations,

(1) EncryptimeABE=f1(tt,nc,cc)

(2) LatencyIoT=f2(rt,pt,dt),

where *EncryptimeABE* = encryption time for ABE, *LatencyIoT* = latency for IoT, *tt* = transmission time taken by attribute from IoT device to ABE system, *nc* = number of attributes related to encrypted data, *cc* = computational overhead of ABE encryption algorithm, *rt* = total round trip time taken for communication between IoT devices and their infrastructure, *pt* = processing time taken by IoT devices to encrypt the data, and *dt* = transmission time taken by data from IoT device to ABE system. The function *f*1 represents the relationship between these factors and the encryption time in ABE. It considers how the transmission time, number of attributes, and computational overhead affect the encryption process. Function *f*2 represents the relationship between the factors affecting latency in IoT. It takes into account the round-trip time, processing time, and data transmission time.

Furthermore, several recent IoT devices use blockchain data structures to store data in Ethereum [70]; the data here are public and easy to attack. ABE considers all data storage to be untrusted, as they are managed by a third party and connected to the internet, which can be vulnerable to cyberattack and hacking. For all of the above reasons, we understand that data encryption is important while it is in storage, and ABE helps to preserve confidentiality and accessibility. In our survey, nodes that are accessible from the perspective of data connection (routers/gateways) are not considered secure connections and can be prevented from accessing data. Therefore, to maintain trust, ABE requires an ‘authority key’ in its architecture for secure access control. The key authority is responsible for key management activities such as key creation, deletion, updating, and revocation.

In the previous section, we noted that there are three KPIs and three APIs that the ABE scheme offers to an IoT application. The choice of key and performance indicators for an application is debatable and should be analyzed accordingly. The CPU efficacy of the data generator is important for applications related to IoT, as data are generated by devices that have very limited processing abilities. Most hardware used in IoT environments is CPU limited, such as ESP32 [71] and the Zolertia Re-Mote platform [72]. These devices are often battery powered; therefore, communication protocols used by them should have low bit rates, e.g., Bluetooth, NB-IoT, and LoRa. This makes the bandwidth effectiveness of the data generator a key indicator. It is found that, at the user end, the CPU and bandwidth efficiency are not of much importance, as users with resourceful devices are typically the ones who consume data. On the other hand, the storage capacity of the data generator is considered one of the APIs because ABE takes a very small amount of storage. In the event a scheme requires more storage, there are techniques to alleviate it. Note that if on-board storage is the only option, then the storage ability of the data generator may represent a major constraint for total system efficiency. This is due to the fact that a large amount of coded data is stored locally; this makes the storage ability of the data generator a KPI in some specific cases. For the authority key, CPU efficacy is not considered KPI, but its bandwidth effectiveness is one of the KPIs because of its scalability issues. It is a well-known fact that the traffic from authority keys will increase if the number of users increases. According to IoT analytics research, in 2021, there were approximately 12.3 B IoT networks throughout the world, which may rise to 27.1 B by 2025.

In general, users should choose the most important key performance indicator for a given IoT application before moving on to the next step. They should then choose a group of ABEs that perform well on that criterion. Then, the second-most essential indicator should be chosen and, similarly, the procedure continues until it narrows down the scheme options.

## 6. ABE Implementation and Performance Factors

An attribute is the essential component of ABE, associated with data or a data user. The access authorization connected to these attributes is described by an access policy (AP). It is commonly expressed using a Boolean formula with arguments. An access policy can be represented as a tree where the leaf nodes represent attributes, and where the intermediate nodes represent Boolean operators.

**Figure 4:**
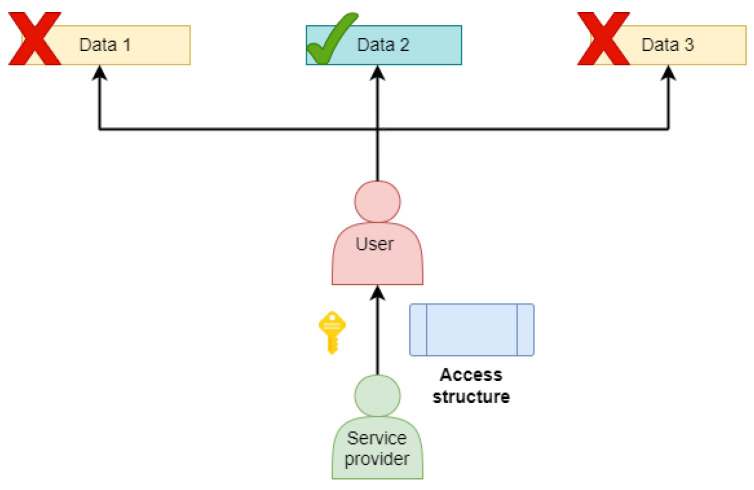
Diagram of KP-ABE scheme.

**Figure 5:**
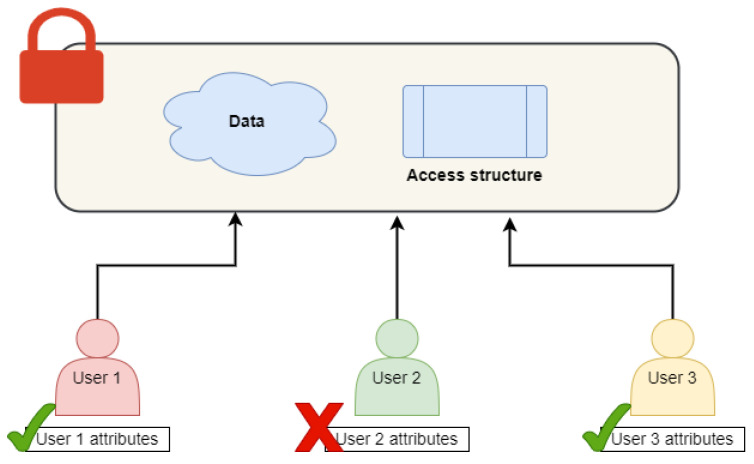
Diagram of the CP-ABE scheme.

ABE is broadly classified into two categories: key policy attribute-based encryption (KP-ABE) and ciphertext policy attribute-based encryption (CP-ABE). Both cases require a copy of the public parameters that are available to everyone who wants to encrypt data and are unique to each party. In addition to this, a secret individual decryption key for each decrypting party is required in order to decrypt data. In KP-ABE, decryption keys are linked to an AP and ciphertexts to a collection of characteristics that define them. APs describe the “capacity to access what”, referred to as the decryption key owner. Since KP-ABE schemes choose access authorizations when generating decryption keys, they empower the authority key. On the other hand, in CP-ABE, decryption keys are linked to a set of attributes, and ciphertexts are linked to AP. The ”ability to be accessed by whom”, referred to as encrypted data, is described by APs. As access authorization is chosen at the time of data encryption, CP-ABE systems give data generators more control. Both KP-ABE and CB-ABE are represented in Figure 4 and Figure 5.

The algorithms implemented by any KP-ABE scheme are as follows: 1. (MK, P) = Setup (Sec). This approach generates a random master key MK and the public parameters represented by P, initializing the scheme with a strength determined by the security parameter Sec and returning them. Whereas the public parameters are made available to the general public and are used to encrypt the data, the master key is kept private by the authority key. 2. C = Enc (Msg, a, P). This encrypts the plaintext message Msg defined by an attribute set using the public parameter P. The outcome is an encrypted message C having an attribute set. 3. DK = Key (AP, MK). Here, using the master key, a new decryption key DK related to the access policy AP is generated. 4. Msg = Dec (C, DK). With the help of the DK, this algorithm decrypts ciphertext C. If AP = true for the set of attributes included in C, then the original message is returned; otherwise, it returns null.

For CP-ABE, the algorithm is implemented as follows: 1. (MK, P) = Setup (Sec). It is similar to the one in KP-ABE. 2. C = Enc (Msg, AP, P). Using the public parameter P, this encrypts a message related to the access policy. It then sends the encrypted message back with the specified access policy included. 3. DK = Key (a, MK). Create a decryption key related to the attribute set using a master key. 4. Msg = Dec (C, DK). With the help of DK, this algorithm decrypts the ciphertext. If AP = true for the attribute set included in the DK, then the original message is returned; otherwise, it returns null.

Another important term in the ABE scheme is “attribute universe”, which is a collection of all attributes in APs or attribute sets. An ABE scheme is typically categorized into (1) small-universe and (2) large-universe schemes. In a small-universe scheme, the authority key, or the entity that runs the setup algorithm, is required to set a limit on the attribute universe at setup time. Public parameters in small-universe methods increase linearly as the size of the attribute universe increases. After setup, the authority key often has the ability to add new attributes, but at the same time, the public parameters should also be updated and forwarded to all data generators. While in a large universe, the authority key does not set a finite attribute universe during setup. Public parameters in large-universe methods have fixed sizes independent of the attribute universe, insofar as data generators are free to add new characteristics whenever they want without consulting the key authority.

Practically, an ABE scheme must have more key management functionalities, such as offering procedures for distributing and revoking decryption keys. Key revocation is considered to be more difficult than key distribution, which is typically simple in cases where a new customer is involved [21,73,74,75,76]. A non-revocable scheme can be changed to a revocable one by following some steps as follows. To revoke a key, the setup algorithm is used by the authority key to generate a new master key and set of public parameters. The authority key then creates a new DK for each nonrevoked consumer with prior access privileges. After that, the authority key has two choices for secretly distributing the new decryption keys. With each customer, it can create a secure channel, for instance, using DTLS (datagram transport layer security). If not, it can use the user’s public keys, such as RSA, to encrypt new decryption keys and then store the resulting ciphertexts in the data storage. The second method is preferred, as it concludes revocation rapidly. Therefore, upon data requests from consumers, the key distribution operation is lazily assigned to the data storage and carried out. It is to be noted that if there are multiple key revocations by the consumer, then only multiple decryption keys will be sent. The updated public parameters are kept in the data storage by the authority key. The most recent public parameters should be extracted by the producers from the data storage before encrypting a new section of the data. This type of revocation mechanism is known as naive revocation, as it works for all nonrevocable schemes.

### 6.1. CPU Efficacy of Data Generator

The CPU load is known to depend on the encryption operation performed at the end of the data generator. Data generators are also involved in the key management process, but they only need new parameters or a list of users to be downloaded whose access has been revoked instead of performing any computation. A large-universe ABE scheme uses hash functions that are less performant than the typical small-universe scheme, as their output is elliptical curves, which are heavy for resource-constrained devices. Therefore, a small-universe scheme can be used to save the CPU efficacy of the data generator.

Using a digital envelope allows data generators to encrypt a symmetric key, with ABE having a specified attribute set (in the case of KP-ABE) or a certain policy (in the case of CP-ABE), which is another straightforward method used in [75] to reduce the CPU burden of the data generator. After this, plaintext is encrypted using a symmetric key to protect it from attribute sets or policies. This method can be used with any ABE scheme and is helpful when a data generator needs to encrypt it multiple times with the same attribute set or policy. As symmetric keys are required to be retained by both the data generator and user, and also possibly need to be revoked by the authority key, this complicates the key management process. Along with these techniques, there are three more approaches that can be used to improve the CPU efficacy of data generators: encryption outsourcing, adopting mathematical alternatives, and Type III pairing.

1. Encryption outsourcing: This requires certain specifications to be fulfilled in order to reduce the CPU load of the data generator, such as fully resourceful neighbors or the existence of users who, on a regular basis, load the data generator with precalculated quantities. Touati et al. [77] propose a CP-ABE scheme for message encryption. To accomplish this, the data generator must first create secure links with at least two reliable full-resource neighbors to whom they can assign difficult tasks. The generator combines the partial results that the neighbors compute and sends them to create the final ciphertext. The authors of [78] advocate a similar KP-ABE approach. Both this and the above offloading schemes considerably reduce the strain on the data generator, but they require a number of resourceful devices in the neighborhood that may not always be there. Second, because of the outsourcing system’s bandwidth impact, generators may end up spending more time and effort communicating than processing. Hohenberger and Waters [79] propose a KP-ABE and CB-ABE scheme in which encryption is done in two phases. In the first ’offline’ phase, the preprocessing of difficult operations takes place, and in the second ’online’ phase, the ciphertext is generated using lighter operations. This is useful in cases where the data generator requires battery charging, as in mobile devices (smartphones). In conclusion, outsourcing encryption in IoT is not generally applicable, but if there are reliable full-resource devices near the data generator, outsourcing is feasible. This occurs in the case of one or more full resource gateways in a network of data generators managed by a unique entity. Therefore, these full-resource nodes act as a point of trust for the entire system, and their compromise might impact data confidentiality.

2. Adopting mathematical alternatives: ECC [80,81] and RSA [82] are known to be pair-free and the fastest techniques that some ABE schemes can use. In fact, RSA-based techniques use basic mathematics that can easily be accelerated by hardware in IoT devices [73]. However, ECC curves are often found to be more efficient than pairing-friendly curves having the same level of security. This is due to the fact that they can be expressed using the fewest number of bits, such as 160 bits for obtaining 80-bit security. The pairing-free ABE schemes [80,81], which use ECC calculations, and [82], a study that uses RSA calculations, are well-known in the literature.

3. Type III pairing: We can accelerate cryptographic procedures by employing Type III pairings instead of Type I pairings as they allow for faster operations on components by allowing smaller representations of elements with the same security level [83]. A large number of ABE protocols already in use, such as [9,23,84], can be easily ported to Type III pairings. However, there are formal procedures to change a security proof with Type I pairings to an equal one with Type III pairings [85]. Adopting this pairing may not be a practical solution in IoT applications when the CPU efficiency of users and/or the key CPU efficiency of the authority are more crucial than data generators. Some ABE schemes, such as [83,86], have been specifically designed for Type III pairings to increase the efficiency of encryption procedures.

### 6.2. Bandwidth Effectiveness of Authority Key

The bandwidth effectiveness of the authority key depends on key management activities. Systems that are set up in such a way that they need to be operated over a long period of time should be aware that users’ roles and privileges change with time, that users can join or leave the system anytime, and that the users’ keys may be compromised (theft or attacked). Therefore, in such cases, it is the responsibility of the authority key to provide new keys or revoke the old ones. For a joining consumer, key revocation is more difficult than key distribution. Key revocation can be categorized into three categories: direct, indirect, and attribute-wise. Now, we discuss different solutions for handling key revocations that have been put forth in the literature and discuss others that are already in use.

Three main approaches have been identified to help reduce the traffic of the authority key: (i) direct revocation that completely relieves the authority key of the key revocation tasks [87,88]; (ii) implementing binary trees in indirect revocation, which transforms the user’s traffic on the authority key from linear to logarithmic [89,90,91,92,93,94]; and (iii) attribute-wise revocation, which bases the amount of traffic produced by revocation jobs on the number of revoked attributes rather than on the number of customers [95]. It also has the advantage of keeping the authority key offline and not performing any task until a revocation takes place. Furthermore, since attribute-wise revocation is not dependent on time, a key revocation may take place right away.

### 6.3. Bandwidth Effectiveness of Data Generator

The encryption bandwidth overhead (difference in the size of ciphertext and plaintext) and the key management bandwidth overhead (key distribution and key revocation traffic) are two examples of the bandwidth overhead that a scheme imposes on the data generator. Using the digital envelope approach is a common and easy solution to reduce the encryption bandwidth overhead, as it uses symmetric-key encryption that has significantly less encryption bandwidth than ABE does; however, it makes key management a more complex process. In addition to this, three key methods to reduce the producer’s bandwidth overhead include: (i) fixed-size ciphertext; (ii) using a reliable key management system; and (iii) using small group elements for the ciphertexts and public parameters [83].

1. Fixed-size ciphertext: Any overhead in encryption bandwidth can be reduced by using ciphertexts of small or fixed size. Generally, the number of attributes in KP-ABE or CP-ABE determines the ciphertext size in many ABE systems. It is obvious that this dependency is not good for the producer’s traffic. Therefore, a fixed-size ciphertext helps to reduce the dependency and data traffic. Fixed-size ciphertext schemes use access structure languages that are not very expressive, whereas variable-size ciphertext schemes use access structure languages that are more expressive, enabling the development of APs that are not possible in fixed-size ciphertext schemes [96,97,98]. Ref. [99] also contains ciphertext of the smallest size and is very poorly expressive.

2. Using a reliable key management system: Direct, indirect, and attribute-wise revocation are the three different key revocation procedures. A mechanism with the most consistently minimal effect on the producer bandwidth is what we are trying to find. An attribute-wise revocation scheme that is not reliable for the data generator is discussed in [74]. On the other hand, in schemes such as [95,98], there is no need for the producers to download anything following a revocation. Therefore, to enforce the revocation, a producer should always encrypt data using the same public parameters before uploading the ciphertext to the data store. In direct revocation, the data generator often needs to keep a list of the user identifiers who had their access revoked. Usually, generators only incur this expense in terms of bandwidth for important managerial processes. More data generators will be denied access while the system operates, but downloading the whole list of revoked users each time could be harmful to the data generator’s bandwidth and its low storage capacity. To reduce the generator’s key management bandwidth overhead, the direct revocable technique [88] removes expired decryption keys from the revocation list. Lastly, the data generator may not need to download anything when using the indirect revocation procedure. In [89,100], the data generator contributes to revocation by adding extra attributes to the ciphertext at the time of encryption. Thus, the bandwidth requirement is saved by requiring no communication with the other parties.

3. Using small group elements for the ciphertexts and public parameters: The bandwidth overhead caused by the ciphertext’s size can be decreased by using small group elements. Making the most G elements in the ciphertext into G1 elements is a practical way to modify ABE schemes to adopt Type III pairings. In addition to saving the data generator’s bandwidth, smaller G1 elements make it easy to compute some encryption procedures more effectively. On comparing a ciphertext of [23] to that [83], we find that encryption bandwidth overhead is reduced to 53.60% for each ciphertext uploaded to the data store.

## 7. Opportunities and Challenges of ABE in IoT

ABE presents various opportunities to provide security in the IoT. It provides fine-grained access control, which is particularly relevant in IoT environments that require dynamic and flexible access control policies. For example, in a smart home, different users may have different access levels and capabilities that may change over time. ABE grants or revokes access based on changing circumstances, such as the user’s location or role, without compromising the system’s security. It has the ability to improve the confidentiality and privacy of data. In the case of an IoT environment, devices collect sensitive data that must be protected from unauthorized access. ABE helps to protect these data by allowing them to be encrypted with attributes, such as the user’s location or identity. Only users who have the necessary attributes can decrypt the data, so if an attacker were to gain access to the encrypted data, they would be unable to decrypt it without possessing the required attributes. Moreover, ABE can offer a scalable and effective solution for handling access control policies in IoT deployments at a large scale. By using attributes, ABE enables the definition of access policies that can be managed and updated in a centralized manner. This can be particularly useful in IoT environments that involve a large number of devices with different capabilities and access levels.

The adaptability and dynamic nature of ABE in IoT applications present another potential. ABE allows dynamic attribute-based access control policies that allow for efficient data exchange and collaboration in various IoT scenarios. IoT devices usually operate in dynamic situations where attributes change frequently; therefore, ABE gives users the flexibility to manage and enforce these dynamic access control settings, ensuring data security. Furthermore, ABE provides the framework for data-centric IoT security and privacy. It enables the direct application of security and privacy regulations to the data without concern for the underlying infrastructure or devices. Even in highly distributed and heterogeneous IoT environments, this approach enables IoT devices to apply access control policies and ensure data protection. In an IoT environment, ABE enables safe data aggregation and collaboration. As in the IoT, numerous devices produce data that must be collected and analyzed as a whole. ABE enables attribute-based encryption of data from various devices, allowing for decryption and aggregation of the data while maintaining privacy. This encourages collaborative data analysis and decision-making in IoT applications, resulting in better insights and results. All these opportunities discussed above encourage us to use ABE in an IoT environment.

Implementing ABE in IoT also comes with some challenges. First, there is a significant computational overhead associated with ABE, which can be a challenge in resource-constrained IoT devices. ABE requires complex cryptographic operations, such as bilinear pairings and attribute-based access policy evaluation, which can be computationally expensive for IoT devices with limited processing power and memory. Second, interoperability can be a challenge when implementing ABE in IoT environments. Different IoT devices may use different communication protocols and data formats, making it difficult to implement a standardized ABE system that can work with all devices. Ensuring compatibility between devices is essential for the successful implementation of ABE in IoT. Third, managing attribute authorities and attribute revocation can also be challenging in IoT environments. Since IoT devices are highly dynamic and may frequently join or leave the network, managing the attributes associated with each device can be challenging. In addition, revocation of attributes when devices are lost, stolen, or no longer trusted can be complex, and attribute revocation mechanisms must be carefully designed to avoid compromising system security. Scalability is another challenge facing ABE when deployed in a large-scale IoT environment. As the number of IoT devices increases, the complexity of establishing and implementing access policies for each IoT device becomes an overhead. Complex cryptographic processes are frequently involved in ABE, and scaling these operations to support a large number of devices can impose a strain on computer resources and lead to latency and increased response times. Ensuring the privacy of user attributes can also be a challenge in the implementation of ABE in the IoT. Attributes can contain sensitive information and, if not properly managed, could be used to identify users or reveal sensitive information about them. Careful consideration should be given to how attributes are generated, stored, and shared to ensure that users’ privacy is protected. Lastly, to encourage the usage and compatibility of ABE in IoT, interoperability and standardization challenges must be resolved. Enhancing interoperability and enabling secure and effective communication between heterogeneous IoT settings can be achieved by creating standardized protocols, frameworks, and interfaces that make it simple to integrate ABE into various IoT devices and platforms.

**Figure 6:**
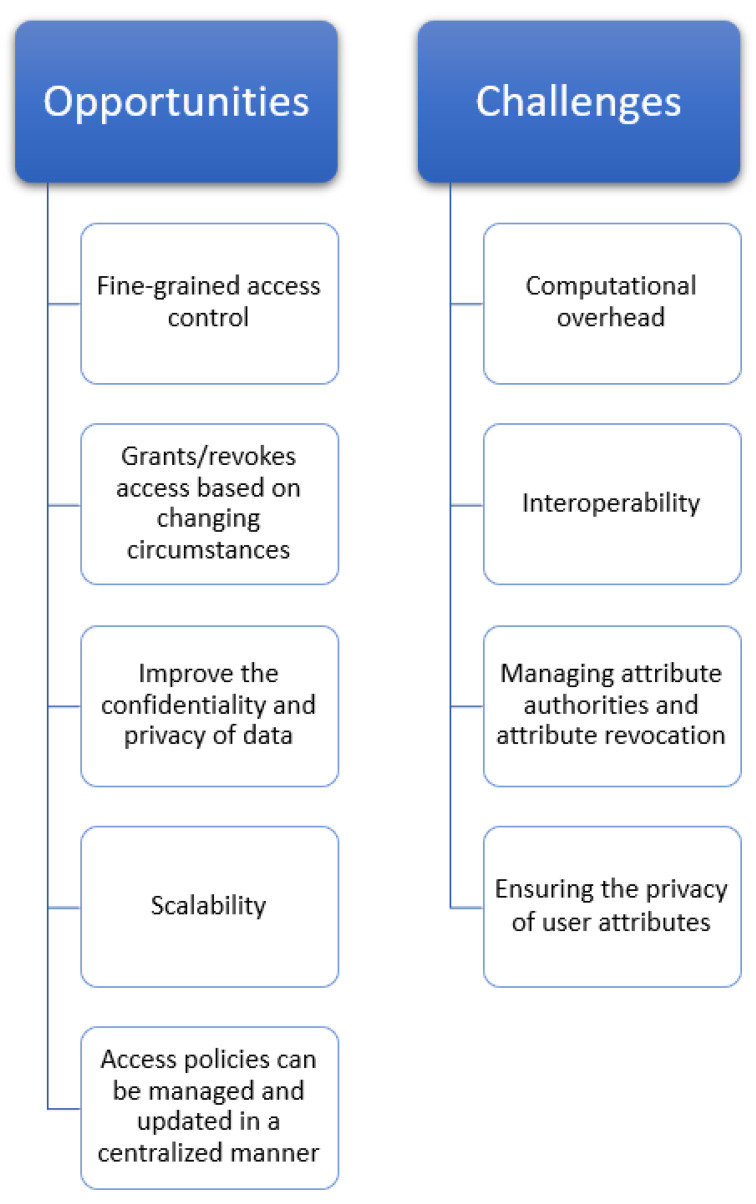
Opportunities and challenges faced by ABE in IoT.

Addressing these challenges is crucial for the successful implementation of ABE in IoT environments. However, with proper design and implementation, ABE can provide an efficient and secure way to manage access control and enhance data confidentiality and privacy in the IoT. Figure 6 shows various opportunities and challenges faced in implementing ABE in an IoT environment.

## 8. Evaluation

In this section, we analyze the performance of some ABE schemes that were discussed in the previous sections. A comparative analysis of this kind helps clarify performance-related issues and gives us more clarity on how beneficial each ABE scheme is. The schemes are selected from both KP-ABE [74,79,83,89,90] and CP-ABE [79,83,88,99,100]. Each scheme performs well in 1 or 2 KPIs, but not in all 3, as shown in Table 5. Indirect key revocation and effective key management are implemented in [89,100]. In Ref. [83], (i) and (v) use small group elements in ciphertexts and Type III pairings. The attribute-wise revocation is implemented in [74], and a hybrid revocation in [90]. Ref. [79] uses encryption offload to lessen the computational burden on data generators. Direct key revocation and effective key management are implemented in [88,99], consisting of the smallest fixed ciphertext.

The schemes under observation have authority keys, data storage, data generators, and data users that generate events such as data production and consumption, user joining, and revocation of keys when simulated by the simulator. These events simulate the relevant algorithm for each scheme, such as encrypt, decrypt, etc. The simulator does not implement any messaging mechanism or mathematical operations; instead, it keeps track of numbers and kinds of mathematical operations to estimate the overall computational load. The simulator also calculates the traffic overhead and records the amount and size of messages sent and received between entities. The architecture in Figure 3 is studied here. The universe is defined by the simulator using some specific number of attributes for each scheme considered. The shape of the access policy remains constant throughout the simulation for all ciphertexts and decryption keys. The simulator can be set up to have a specific number of data generators and users at the beginning. The number of users changes each time a user joins or key revocation takes place, whereas the number of generators stays constant during the simulation. According to the schemes above, in the initial stage, the simulator generates a database of ciphertexts and decryption keys for the users. We ensure that every decryption key has the ability to open at least one of the initial ciphertexts while generating decryption keys. After initiation, the simulator begins to create events (producing data, consuming data, consumer joining, and key revocation) and to log metrics.

For every scheme, the data generator encrypts the new data at their creation and uploads the ciphertext to storage. For each data consumption, a random user downloads and decrypts a random ciphertext from storage. For each scheme, the authority key will remove a random user from the system at each key revocation event. Similarly, at each user joining event, the authority key creates a new decryption key for each scheme, encrypts it using the user’s public key, and uploads it to storage. The data user then downloads and decrypts the key. These actions are taken up to the end of the simulation period. The results per generator and user are determined in the final stage. We consider all generators to be IoT hardware. We conduct benchmarks on such a device using the PBC library to find out how long different basic math operations take to complete.

In [90], data generators can decide whether to encrypt in a ’direct’ or ’indirect’ revocation mode. We simulated two variations of the [90] scheme and used a random decision during data creation to model this opportunity, i.e., hybrid and direct. In a hybrid, the chance of creating a ciphertext was set to half, while in direct variation, the scheme is forced to function as a pure direct revocation by setting the probability equal to 1. Since the pure indirect variation is almost similar to [89], we ignore it. The time duration is set to 1 day for simulation, so in the end, the authority key generates a key update and stores it in storage. In our simulations for [79], (i) and (ii), we assume that the ’offline phase’ is assigned to a reliable device, and the resulting pre-processed quantities are then sent to data generators via a secure connection. After the completion of the ’online phase’, the data generator uploads the ciphertext to storage. Only access policies made up of the AND gate on Boolean attributes that also use wildcards are allowed by [99]. For comparison, the redundancy method is generally used to increase the expressiveness of the scheme.

## 9. Discussion and Results

**Figure 7:**
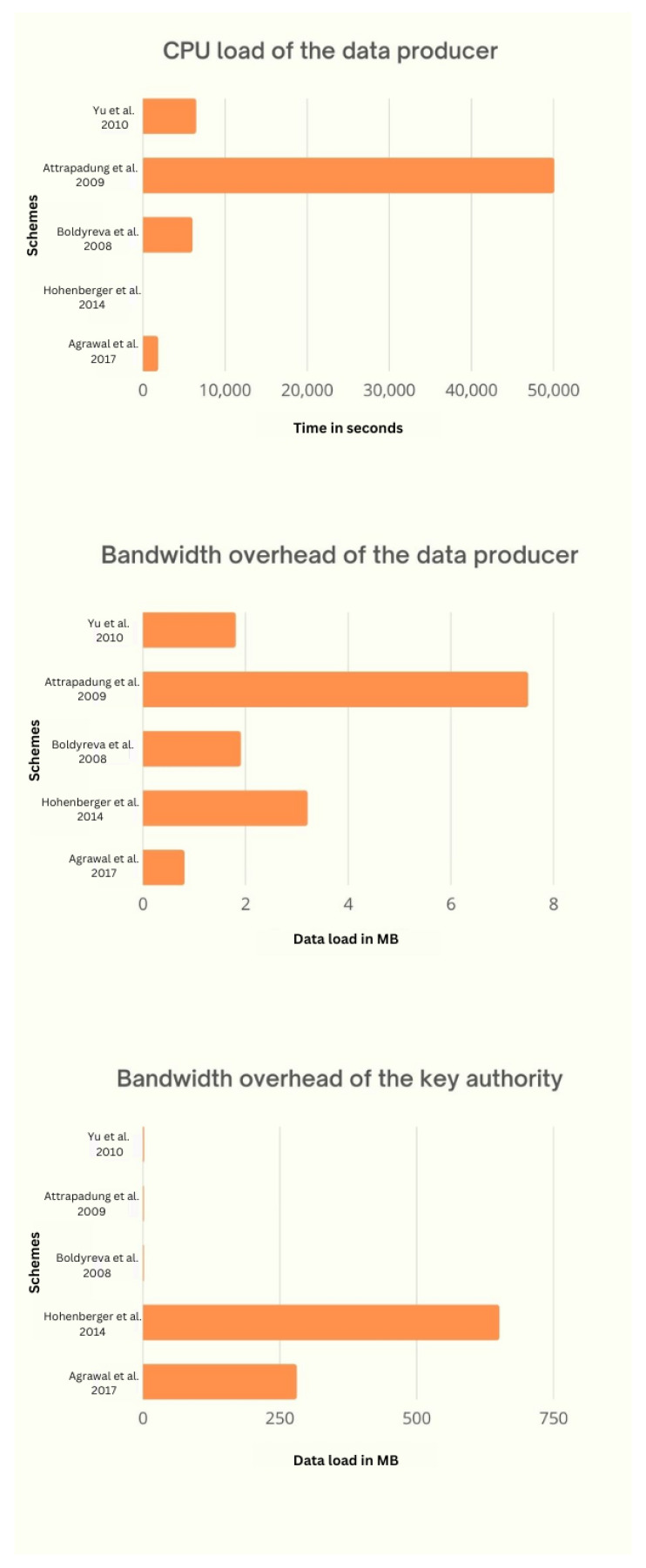
Comparison of various KP-ABE schemes with performance indicators, where Yu et al. 2010—[74], Attrapadung et al. 2009—[90], Boldyreva et al. 2008—[89], Hohenberger et al. 2014—[79] and Agrawal et al. 2017—[83].

In this section, the performance of various ABE schemes is discussed versus KPIs. Both KP-ABE and CP-ABE are separately analyzed because they are not implicitly comparable. Firstly, as we observe the results of KP-ABE schemes, it is found that the best scheme among others in comparison to the performance of the data producer is [83], as it minimizes bandwidth and CPU overhead by utilizing asymmetric pairing (Figure 7). The schemes [74,89] are less efficient than [83], but in the case of handling key revocation, [74,89] are found to be better and more efficient than [83]. The revocable [90] method only functions effectively in terms of the effectiveness of the authority key bandwidth. In fact, the data generator is less efficient when using direct revocation than when using indirect revocation, since it has to perform more calculations and produce longer ciphertexts. In Ref. [79], (i) uses encryption outsourcing to relieve the data generator of difficult tasks. It only performs a small number of time-efficient multiplications. However, downloading the pre-processed quantities will negatively affect the data generator’s bandwidth efficiency. Furthermore, the simple revocation in [79], (i) has a significant bandwidth overhead on the authority key. Table 6 compares various KP-ABE schemes with their performance indicators.

**Figure 8:**
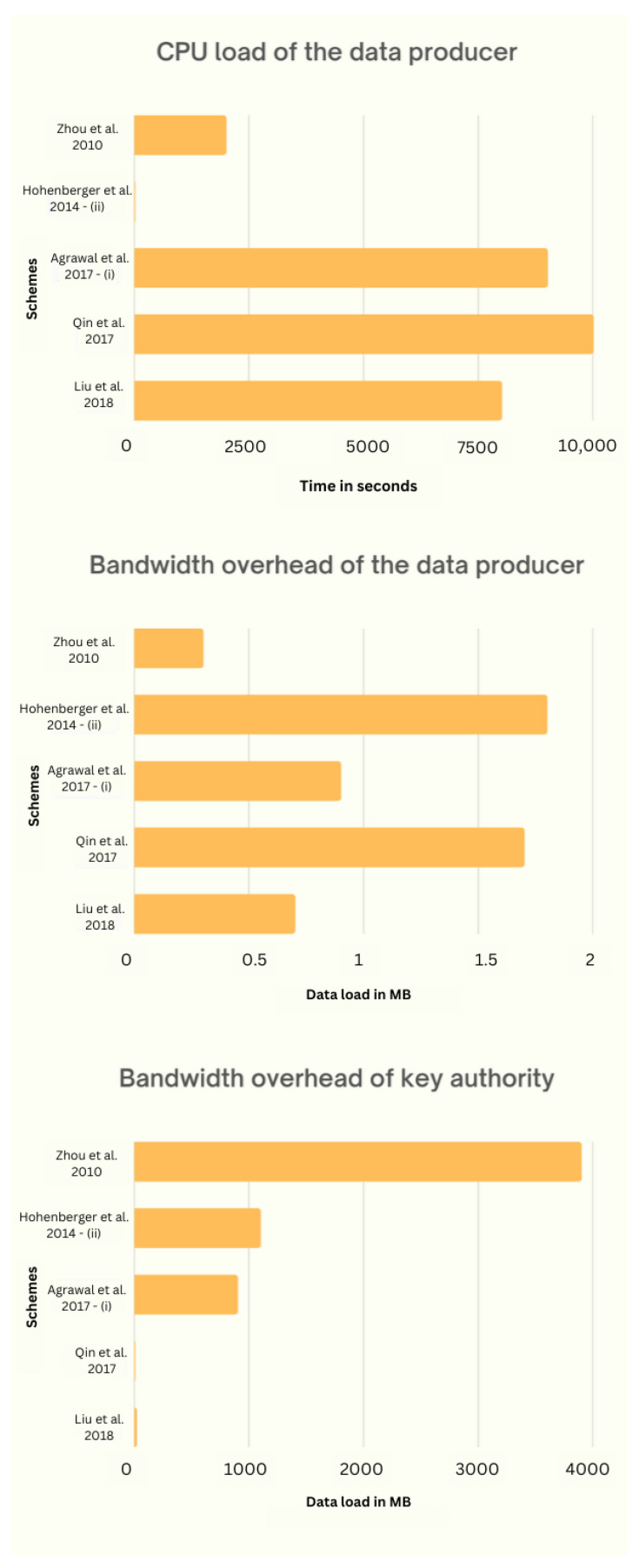
Comparison of various CP-ABE schemes with performance indicators, where Zhou et al. 2010—[99], Hohenberger et al. 2014—[79], Agrawal et al. 2017—[83], Qin et al. 2017—[100] and Liu et al. 2018—[88].

CP-ABE schemes are also observed for their KPIs, as shown in Figure 8. We observe that, for [79], (ii), the CPU load of the data generator is exceptionally less, compared to [79], (i). The simulated asymmetric painting scheme of [83], (i) does not have the lowest computing load for the generator, in contrast to the prior simulation. The performance of this approach is significantly impacted by the computation of the hashes, which are not pre-computable. As a result, Ref. [83], (i) has very high encryption efficiency. It has been shown that, despite being incredibly efficient on PCs, it loses a significant amount of efficiency when used in IoT applications. In CP-ABE, [99] is found to have the best CPU and better bandwidth effectiveness of the data generator. However, in the case of ignorant revocation and bandwidth effectiveness of the authority key, Ref. [99] is the worst. The bandwidth overhead of the key authority is minimal for the revocable schemes [88,100]. The scheme [88] is more effective for the data producer, despite the fact that it uses a direct revocation and requires the data generator to carry out more computations to carry out the revocation. The data generator faces a slight increase in CPU and bandwidth overhead [100]. This approach is well-suited for applications where a high level of data consumer efficiency is required. Table 7 compares various CP-ABE schemes with their performance indicators.

Moving on to APIs, there are three ways to increase the storage ability of data generators: first, by using large-universe; second, by partially storing the public parameters, and third, by using key revocation methods that are efficient for storage. Data generator storage may be affected by the revocation method. In direct revocation, the revoked identifiers are contained in the revocation list, which each data generator must keep. Therefore, the use of an indirect revocation usually results in higher generator storage efficiency [88]. The computational efficiency of data users in ABE depends on the operations performed during data decryption and key management. Data decryption efficiency can be improved by: first, outsourcing difficult decryption procedures [94,100]; second, using decryption with constant complexity; and third, using the ECC or RSA method. The tasks involved in the data user bandwidth overhead are the same as those in the data generator. Therefore, to improve the bandwidth effectiveness of data users, we require: fixed-size ciphertext [94,100], small group elements, direct key revocation [88], and a part of data storage used for decryption.

## 10. Conclusions and Future Work

In this study, we reviewed appropriate ABE schemes for Internet of Things applications. We looked at different approaches in view of the three KPIs. The strategies used by the most advanced schemes to increase these performance indicators were then identified and documented. Finally, using detailed simulations, we evaluated the effectiveness of a few well-known ABE methods. This survey revealed numerous difficulties in developing an ABE scheme suitable for the Internet of Things. Researchers might look at the various techniques we discussed to enhance the identified KPIs and APIs to address these issues, or they may work to develop new approaches to enhance the suggested performance metrics. Additionally, we found that using IoT devices with hardware accelerators designed specifically for ABE cryptographic processes will greatly boost the system’s overall performance. Additionally, researchers should focus on pairing-based cryptographic hardware accelerators to improve the performance of IoT devices during encryption and decryption. Schemes with extra features always incur additional expenses and are sometimes difficult to implement in an IoT environment. However, these extra features are desirable for cryptography, and research should focus on enhancing existing schemes and introducing new ones that are capable of reducing the computation and communication overhead of data producers.

### Future Work

In the future, several areas can be improved for use of ABE in IoT environments. One area is the development of more efficient ABE algorithms, which can reduce computational overhead and make it more feasible for resource-constrained IoT devices. ABE can also be integrated with other security mechanisms, such as blockchain [101], to enhance the overall security of IoT systems. Another area of focus is the development of ABE-based access control frameworks that can be used in different IoT domains, such as smart homes, smart cities, and industrial IoT. The work can also be extended by adding concepts such as deep learning and artificial intelligence, which will train the machine to protect the system against security threats and will help to increase overall accuracy and robustness [102]. By addressing these areas of focus, the use of ABE can be enhanced and optimized for secure and efficient IoT deployments. Moreover, quantum-resistant ABE techniques are currently impractical for IoT devices due to the large size of the encryption and decryption keys. As a suggestion, researchers should focus on developing more effective quantum-resistant strategies for ABE, as stakeholders will have access to quantum computers in the next few decades.

## Figures and Tables

**Table 1:** Comparative analysis between traditional cryptography and ABE.

Criteria	Traditional Cryptography	ABE Scheme
Access control	Limited	Flexible
Key management	Centralized	Decentralized
Data sharing	Challenging	Seamless and efficient
Fine-grained control	Limited	Highly customizable
Attribute-based control	Not supported	Supported
Scalability	Moderate	Scalable
User revocation	Complex and costly	Supported and efficient
Privacy	Limited	Enhanced
Complexity	Low	High
Performance	High overhead	Variable and depends on the domain of implementation
Use case	General	Data-centric access control environment

**Table 2:** Benefits and drawbacks of ABE.

Benefits	Drawbacks
Fine-grained access control	Complexity in key management and policy definition
Data confidentiality and privacy	Increased computational overhead
Flexible and scalable access policies	Dependency on attribute authorities and attribute revocation
Computational overhead	High
Support for dynamic environments	Potential for collusion attacks
Collaboration and sharing of encrypted data	Limited standardization and interoperability
Reduces the need for trusted third parties	Potential for increased ciphertext size
Protects against insider threats	Additional complexity in implementation and deployment

**Table 3:** Comparison between different types of ABE schemes.

Criteria	KPABE	CPABE	MABE	HABE
Efficiency	Average	Average	Flexible	Better than others
Access control	Low	Average	Average	High
User accountability	Yes	Yes	Yes	No
Computational overhead	High	Average	Higher than others	Higher than others
Support for fine-grained access control	Yes	Yes	Yes	Yes
Limitation	No ability to decide who can encrypt data	Decryption key supports only user attributes that are logically organized	Every authority attribute set should be disjoint	Not suitable for implementation
Confidentiality of data	No	Yes	Yes	Yes
Resistance to collusion	Yes	Yes	Yes	Yes
Components included	Data are related with an access policy	Ciphertext is related with an access policy	Hierarchical generation of key	Multiple authorities are there

**Table 4:** Recent works using ABE.

Paper Reference	Year	Scheme	Domain	Advantage	Limitation
Tan et al. [60]	2019	Hierarchical KP-ABE (H-KP-ABE)	IoT	Suitable for IoT-based applications having low-powered devices.	The design of the security proof in a stronger security model is an open problem.
Yang et al. [61]	2020	CP-ABE scheme having constant size ciphertexts and being fully secured with Type III bilinear pairing and the access structure of the AND gate.	IoT	Constant computation overhead for decryption and is independent of the number of attributes involved.	Works only with constant-sized ciphertext and can be applied only in a large universe.
Han et al. [62]	2020	A traceable and revocable attribute-based ciphertext policy encryption (TR-AP-CPABE)	Binary tree associated with user information	Efficient, secure, and provides selective access policy based on the decisional BDHE assumption model.	Lacks traceable and revocable CP-ABE based on the full hidden policy that provides more security.
Sowjanya et al. [63]	2020	A secure framework for a wireless body area network using CP-ABE based on elliptic curve cryptography without bilinear pairing operations.	Wireless body area networks (WBANs)	Better than existing schemes in terms of keys-ciphertext size and computation overhead.	Multi-authority and attribute revocation are not considered.
Ali et al. [64]	2020	A fully distributed revocable ciphertext policy hierarchical ABE (FDR-CP-HABE)	Cloud computing	Efficient, secure, and scalable.	Most of the operations are performed by the cloud.
Wang et al. [65]	2022	Dual-policy attribute-based encryption	Cloud	Due to its flexible access control feature, DPABE is useful in general-purpose applications.	Communication overhead of access structure is ignored.
Hohenberger et al. [66]	2023	Registered ABE	Cloud	Users can generate their own secret key.	Linear registration time.
Saidi et al. [67]	2022	SHARE-ABE	Fog computing	Secure and efficient for resource-constrained IoT devices.	False attribute can be a problem.
Dang et al. [68]	2023	A novel secure video retrieval scheme with ABE and multi-feature fusion	Smart grid	Effectively reduces the overhead of video secure retrieval and improves the retrieval accuracy.	Large and heavy videos cannot result in efficiency.
Wang et al. [69]	2023	PHCA	Smart health	Ensure privacy and security for smart health while maintaining constant decryption cost.	Third-party auditor can be compromised.

**Table 5:** Various works on ABE schemes and the performance indicator they support (the supported indicators are denoted by a checkmark).

Paper Reference	Year	Scheme	CPU Efficacy of Data Generator	Bandwidth Effectiveness of Data Generator	Bandwidth Effectiveness of Authority Key
Boldyreva et al. [89]	2008	Indentity-based encryption	.	✓	✓
Attrapadung et al. [90]	2009	ABE supporting direct/indirect revocation	.	✓	.
Yu et al. [74]	2010	Access control in cloud	.	✓	.
Hohenberger et al. [79]	2014	Online/offline ABE	✓	.	.
Agrawal et al. [83]–(i)	2017	Fast attribute-based message encryption	✓	.	✓
Agrawal et al. [83]–(v)	2017	Fast attribute-based message encryption	✓	.	✓
Qin et al. [100]	2017	Server-aided revocable ABE	.	✓	✓
Zhou et al. [99]	2010	CP-ABE	.	.	✓
Liu et al. [88]	2018	Time-based direct revocable scheme	.	✓	✓

**Table 6:** Comparison between various KP-ABE schemes with their performance indicators.

Performance Indicators	Yu et al. [74]	Attrapadung et al. [90]	Boldyreva et al. [89]	Hohenberger et al. [79]	Agrawal et al. [83]
CPU load of the data producer	Almost the same as [89]	Maximum	Almost the same as [74]	Minimum	Between [79,89]
Bandwidth overhead of the data producer	Almost the same as [89]	Maximum	Almost the same as [74]	More than [74,83,89], but less than that of [90]	Minimum
Bandwidth overhead of key authority	Almost the same as [89,90]	Almost the same as [74,89]	Almost the same as [74,90]	Maximum	Almost half of [79]
Efficiency of handling key revocation	Better than [83]	Average	Better than [83]	Better than [83]	Minimum

**Table 7:** Comparisons between various CP-ABE schemes, with their performance indicators.

Performance Indicators	Zhou et al. [99]	Hoehenberger et al. [79], (ii)	Agrawal et al. [83], (i)	Qin et al. [100]	Liu et al. [88]
CPU load of the data producer	More than [79]	Minimum	Less than [100]	Maximum	Less than [83]
Bandwidth overhead of the data producer	Minimum	Maximum	Average	Little less than [79], (i)	Less than average
Bandwidth overhead of key authority	Maximum	Below average	Less than [79], (i)	Minimum	Minimum

## Data Availability

Not Applicable.

## Abbreviations

The following abbreviations are used in this manuscript:

ABE	Attribute-based encryption
IDS	Intrusion detection system
KP-ABE	Key policy attribute-based encryption
CP-ABE	Ciphertext policy attribute-based encryption
KPI	Key performance indicators
IoT	Internet of Things
ECC	Elliptic curve cryptography
MABE	Multi-authority attribute-based encryption schemes
HABE	Hierarchical attribute-based encryption scheme
AP	Access policy

## References

[1] Yu S., Ren K., Lou W. (2011). FDAC: Toward fine-grained distributed data access control in wireless sensor networks. IEEE Trans. Parallel Distrib. Syst..

[2] Rasori M., Perazzo P., Dini G. (2020). A lightweight and scalable attribute based encryption system for smart cities. Comput. Commun..

[3] Sicari S., Rizzardi A., Dini G., Perazzo P., La Manna M., Coen-Porisini A. (2020). Attribute-based encryption and sticky policies for data access control in a smart home scenario: A comparison on networked smart object middleware. Int. J. Inf. Secur..

[4] Baza M., Nabil M., Lasla N., Fidan K., Mahmoud M., Abdallah M.M. Blockchain-based firmware update scheme tailored for autonomous vehicles. Proceedings of the 2019 IEEE Wireless Communications and Networking Conference, WCNC 2019.

[5] La Manna M., Treccozzi L., Perazzo P., Saponara S., Dini G. (2021). Performance evaluation of attribute-based encryption in automotive embedded platform for secure software over-the-air update. Sensors.

[6] Li Y., Cheng X., Cao D., Wang D., Yang L. (2018). Smart choice for the smart grid: Narrowband internet of things (NB-IoT). IEEE Internet Things.

[7] Kocher P., Horn J., Fogh A., Genkin D., Gruss D., Hass W., Hamburg M., Lipp M., Mangard S., Prescher T. (2020). Spectre attacks: Exploiting speculative execution. Commun. ACM.

[8] Lipp M., Schwarz M., Gruss D., Prescher T., Hass W., Mangard S., Kocher P., Genkin D., Yarom Y., Hamburg M. (2018). Meltdown. arXiv.

[9] Sahai A., Waters B. (2005). Fuzzy identity-based encryption. Annual International Conference on the Theory and Applications of Cryptographic Techniques.

[10] Pardhan B., Singh B., Bhoria A., Singh A.K., Gupta R. (2021). A Comparative Study on Cipher Text Policy Attribute based Encryption Schemes. Int. J. Eng. Res. Technol..

[11] Li X., Gu D., Ren Y., Ding N., Yuan K. (2012). Efficient ciphertextpolicy attribute-based encryption with hidden policy. International Conference on Internet and Distributed Computing Systems.

[12] Oberko P.S.K., Obeng V.H.K.S., Xiong H. (2021). A survey on multi-authority and decentralized attribute-based encryption. J. Ambient. Intell. Humaniz. Comput..

[13] Al-Dahhan R.R., Shi Q., Lee G.M., Kifayat K. (2019). Survey on revocation in ciphertext-policy attribute-based encryption. Sensors.

[14] Edemacu K., Park K.H., Jang B., Kim J.W. (2019). Privacy provision in collaborative e-health with attribute-based encryption: Survey, challenges and future directions. IEEE Access.

[15] Lee C.C., Chung P.S., Hwang M.S. (2013). A survey on attribute-based encryption schemes of access control in cloud environments. Int. J. Netw. Secur..

[16] Balamurugan B., Krishna P.V. (2014). Extensive survey on usage of attribute based encryption in cloud. J. Emerg. Technol. Web Intell..

[17] Moffat S., Hammoudeh M., Hegarty R. A survey on ciphertextpolicy attribute-based encryption (CP-ABE) approaches to data security on mobile devices and its application to IoT. Proceedings of the International Conference on Future Networks and Distributed Systems.

[18] Pang L., Yang J., Jiang Z. (2014). A survey of research progress and development tendency of attribute-based encryption. Sci. World J..

[19] Qiao Z., Liang S., Davis S., Jiang H. Survey of attribute-based encryption. Proceedings of the 15th IEEE/ACIS International Conference on Software Engineering, Artificial Intelligence, Networking and Parallel/Distributed Computing (SNPD).

[20] Zhang Y., Deng R.H., Xu S., Sun J., Li Q., Zheng D. (2020). Attribute based encryption for cloud computing access control: A survey. ACM Comput. Surv..

[21] Ullah S., Zheng J., Din N., Hussain M.T., Ullah F., Yousaf M. (2023). Elliptic Curve Cryptography; Applications, challenges, recent advances, and future trends: A comprehensive survey. Computer Sci. Rev..

[22] Li J., Yu Q., Zhang Y. (2019). Hierarchical attribute-based encryption with continuous leakage-resilience. Inf. Sci..

[23] Chen C., Wang T., Tian J. (2013). Improving timing attack on RSA-CRT via error detection and correction strategy. Inf. Sci..

[24] Osvik D.A., Shamir A., Tromer E. (2006). Cache attacks and countermeasures: The case of AES. Proceedings of the Topics in Cryptology–CT-RSA 2006: The Cryptographers’ Track at the RSA Conference 2006.

[25] Kaur S., Singh B., Kaur H. (2021). Stratification of hardware attacks: Side channel attacks and fault injection techniques. SN Comput. Sci..

[26] Dobraunig C., Mennink B., Primas R. Leakage and tamper resilient permutation-based cryptography. Proceedings of the 2022 ACM SIGSAC Conference on Computer and Communications Security.

[27] Vinothkumar A., Anand M., Ravi S. (2016). Attribute Based Encryption (ABE) Algorithm for Searching and Securing Encrypted Data. ARPN J. Eng. Appl. Sci..

[28] Goyal V., Pandey O., Sahai A., Waters B. Attribute based encryption for fine-grained access control of encrypted data. Proceedings of the 13th ACM Conference on Computer and Communications Security–CCS ’06.

[29] Luo W., Ma W. (2018). Efficient and Secure Access Control Scheme in the Standard Model for Vehicular Cloud Computing. IEEE Access.

[30] Khuntia S., Kumar P.S. New Hidden Policy CP-ABE for Big Data Access Control with Privacy-preserving Policy in Cloud Computing. Proceedings of the 2018 9th International Conference on Computing, Communication and Networking Technologies (ICCCNT).

[31] Fu J., Wang N. (2019). A practical attribute-based document collection hierarchical encryption n scheme in cloud computing. IEEE Access.

[32] Li H., Lan C., Fu X., Wang C., Li F., Guo H. (2020). A Secure and Lightweight Fine-Grained Data Sharing Scheme for Mobile Cloud Computing. Sensors.

[33] Alrawais A., Althohaily A.R., Hu C., Xing X., Cheng X. (2017). An Attribute-Based Encryption Scheme to Secure Fog Communications. IEEE Access.

[34] Chase M. (2007). Multi-authority attribute-based encryption. Proceedings of the Theory of Cryptography: 4th Theory of Cryptography Conference, TCC 2007.

[35] Asim M., Ignatenko T., Petkovic M. (2019). Hierarchical Attribute-Based Encryption and Decryption. U.S. Patent.

[36] Aljuhani A., Kumar P., Kumar R., Jolfaei A., Islam A.N. (2022). Fog intelligence for secure smart villages: Architecture, and future challenges. IEEE Consum. Electron. Mag..

[37] Kumar R., Kumar P., Aloqaily M., Aljuhani A. (2022). Deep Learning-based Blockchain for Secure Zero Touch Networks. IEEE Commun. Mag..

[38] Kumar R., Kumar P., Jolfaei A., Islam A.N. An Integrated Framework for Enhancing Security and Privacy in IoT-Based Business Intelligence Applications. Proceedings of the 2023 IEEE International Conference on Consumer Electronics (ICCE).

[39] Sarker A., Kermani M.M., Azarderakhsh R. (2020). Fault detection architectures for inverted binary ring-LWE construction benchmarked on FPGA. IEEE Trans. Circuits Syst. II Express Briefs.

[40] Shahbazi K., Ko S.B. (2023). An Optimized Hardware Implementation of Modular Multiplication of Binary Ring LWE. IEEE Trans. Emerg. Top. Comput..

[41] Mozaffari-Kermani M., Azarderakhsh R., Aghaie A. (2015). Reliable and error detection architectures of Pomaranch for false-alarm-sensitive cryptographic applications. IEEE Trans. Very Large Scale Integr. (VLSI) Syst..

[42] Ahir P., Mozaffari-Kermani M., Azarderakhsh R. (2017). Lightweight architectures for reliable and fault detection Simon and Speck cryptographic algorithms on FPGA. ACM Trans. Embed. Comput. Syst. (TECS).

[43] Canto A.C., Mozaffari-Kermani M., Azarderakhsh R. (2020). Reliable CRC-based error detection constructions for finite field multipliers with applications in cryptography. IEEE Trans. Very Large Scale Integr. (VLSI) Syst..

[44] Mozaffari-Kermani M., Reyhani-Masoleh A. Reliable hardware architectures for the third-round SHA-3 finalist Grostl benchmarked on FPGA platform. Proceedings of the 2011 IEEE International Symposium on Defect and Fault Tolerance in VLSI and Nanotechnology Systems.

[45] Thirumarai Selvi C., Sankarasubramanian R.S., MuthuKrishnan M. (2022). Detection and Diagnosis of Fault Using Light-Weighted Midori Blocks. Proceedings of the Futuristic Communication and Network Technologies: Select Proceedings of VICFCNT 2020.

[46] Aghaie A., Kermani M.M., Azarderakhsh R. (2016). Fault diagnosis schemes for low-energy block cipher Midori benchmarked on FPGA. IEEE Trans. Very Large Scale Integr. (VLSI) Syst..

[47] Aghaie A., Kermani M.M., Azarderakhsh R. Fault diagnosis schemes for secure lightweight cryptographic block cipher RECTANGLE benchmarked on FPGA. Proceedings of the 2016 IEEE International Conference on Electronics, Circuits and Systems (ICECS).

[48] Li M., Zhao D., Tang X., Cheng S., Hu X., Bao L. Hardware Implementation and optimization Design of Lightweight RECTANGLE Algorithm. Proceedings of the 2020 IEEE 9th Joint International Information Technology and Artificial Intelligence Conference (ITAIC).

[49] Tsantikidou K., Sklavos N. (2022). Hardware Limitations of Lightweight Cryptographic Designs for IoT in Healthcare. Cryptography.

[50] Li H., Tang Y., Que Z., Zhang J. (2022). FPGA Accelerated Post-Quantum Cryptography. IEEE Trans. Nanotechnol..

[51] Kumar A., Ottaviani C., Gill S.S., Buyya R. (2022). Securing the future internet of things with post-quantum cryptography. Secur. Priv..

[52] Anastasova M., Azarderakhsh R., Kermani M.M., Beshaj L. (2023). Time-Efficient Finite Field Microarchitecture Design for Curve448 and Ed448 on Cortex-M4. Proceedings of the Information Security and Cryptology–ICISC 2022: 25th International Conference, ICISC 2022.

[53] Bisheh Niasar M., Azarderakhsh R., Kermani M.M. Efficient hardware implementations for elliptic curve cryptography over Curve448. Proceedings of the Progress in Cryptology–INDOCRYPT 2020: 21st International Conference on Cryptology in India.

[54] Faz-Hernández A., López J., Dahab R. (2019). High-performance implementation of elliptic curve cryptography using vector instructions. ACM Trans. Math. Softw. (TOMS).

[55] Genêt A., Kaluđerović N. (2022). Single-trace clustering power analysis of the point-swapping procedure in the three point ladder of cortex-M4 SIKE. Proceedings of the Constructive Side-Channel Analysis and Secure Design: 13th International Workshop, COSADE 2022.

[56] De Feo L., El Mrabet N., Genêt A., Kaluđerović N., de Guertechin N.L., Pontié S., Tasso É. (2022). Sike channels. Cryptol. ePrint Arch..

[57] Anastasova M., Azarderakhsh R., Kermani M.M. (2021). Fast strategies for the implementation of SIKE round 3 on ARM Cortex-M4. IEEE Trans. Circuits Syst. I Regul. Pap..

[58] Anastasova M., Bisheh-Niasar M., Azarderakhsh R., Kermani M.M. (2021). Compressed SIKE Round 3 on ARM Cortex-M4. Proceedings of the Security and Privacy in Communication Networks: 17th EAI International Conference, SecureComm 2021.

[59] Sanal P., Karagoz E., Seo H., Azarderakhsh R., Mozaffari-Kermani M. (2021). Kyber on ARM64: Compact implementations of Kyber on 64-bit ARM Cortex-A processors. Proceedings of the Security and Privacy in Communication Networks: 17th EAI International Conference, SecureComm 2021.

[60] Tan S.Y., Yeow K.W., Hwang S.O. (2019). Enhancement of a lightweight attribute-based encryption scheme for the Internet of Things. IEEE Internet Things J..

[61] Yang W., Wang R., Guan Z., Wu L., Du X., Guizani M. A lightweight attribute based encryption scheme with constant size ciphertext for internet of things. Proceedings of the ICC 2020-2020 IEEE International Conference on Communications (ICC).

[62] Han D., Pan N., Li K.C. (2020). A traceable and revocable ciphertext-policy attribute-based encryption scheme based on privacy protection. IEEE Trans. Dependable Secur. Comput..

[63] Sowjanya K., Dasgupta M. (2020). A ciphertext-policy Attribute based encryption scheme for wireless body area networks based on ECC. J. Inf. Secur. Appl..

[64] Ali M., Mohajeri J., Sadeghi M.R., Liu X. (2020). A fully distributed hierarchical attribute-based encryption scheme. Theor. Comput. Sci..

[65] Wang T., Zhou Y., Ma H., Zhang R. (2022). Enhanced dual-policy attribute-based encryption for secure data sharing in the cloud. Secur. Commun. Netw..

[66] Hohenberger S., Lu G., Waters B., Wu D.J. (2023). Registered attribute-based encryption. Proceedings of the Advances in Cryptology–EUROCRYPT 2023: 42nd Annual International Conference on the Theory and Applications of Cryptographic Techniques.

[67] Saidi A., Nouali O., Amira A. (2022). SHARE-ABE: An efficient and secure data sharing framework based on ciphertext-policy attribute-based encryption and Fog computing. Clust. Comput..

[68] Dang Q., Zhao B., Sun B., Qiu Y., Du C. (2023). A Secure Image-Video Retrieval Scheme with Attribute-Based Encryption and Multi-feature Fusion in Smart Grid. Proceedings of the Science of Cyber Security-SciSec 2022 Workshops: AI-CryptoSec, TA-BC-NFT, and MathSci-Qsafe 2022.

[69] Wang H., Liang J., Ding Y., Tang S., Wang Y. (2023). Ciphertext-policy attribute-based encryption supporting policy-hiding and cloud auditing in smart health. Comput. Stand. Interfaces.

[70] Viriyasitavat W., Anuphaptrirong T., Hoonsopon D. (2019). When blockchain meets internet of things: Characteristics, challenges, and business opportunities. J. Ind. Inf. Integr..

[71] Espressif ESP32 Platform Datasheet. Espressif Systems, Version 4.2. https://bit.ly/2qW8yj1.

[72] Zolertia RE-Mote Platform Datasheet. https://bit.ly/2OkilYY.

[73] Liu W.C., Hsien W.F., Yang C.C., Hwang M.S. (2016). A survey of attribute-based access control with user revocation in cloud data storage. Int. J. Netw. Secur..

[74] Yu S., Wang C., Ren K., Lou W. Achieving secure, scalable, and fine-grained data access control in cloud computing. Proceedings of the 2010 Proceedings IEEE INFOCOM.

[75] La Manna M., Perazzo P., Dini G. (2021). SEA-BREW: A scalable attribute-based encryption revocable scheme for low-bitrate IoT wireless networks. J. Inf. Secur. Appl..

[76] Rasori M., Perazzo P., Dini G., Yu S. (2021). SEA-BREW: Indirect revocable KP-ABE with revocation undoing resistance. IEEE Trans. Serv. Comput..

[77] Touati L., Challal Y., Bouabdallah A. C-CP-ABE: Cooperative ciphertext policy attribute-based encryption for the internet of things. Proceedings of the Advanced Networking Distributed Systems and Applications (INDS), 2014 International Conference on IEEE.

[78] Touati L., Challal Y. Collaborative KP-ABE for cloud-based internet of things applications. Proceedings of the Communications (ICC), 2016 IEEE International Conference on IEEE.

[79] Hohenberger S., Waters B. (2014). SOnline/Offline Attribute-Based Encryption. Cryptology ePrint Archive.

[80] Yao X., Chen Z., Tian Y. (2015). A lightweight attribute-based encryption scheme for the internet of things. Future Gener. Comput. Syst..

[81] Odelu V., Das A.K. (2016). Design of a new CP-ABE with constant-size secret keys for lightweight devices using elliptic curve cryptography. Secur. Commun. Netw..

[82] Odelu V., Das A.K., Khan M.K., Choo K.K.R., Jo M. (2017). Expressive CP-ABE scheme for mobile devices in IoT satisfying constant-size keys and ciphertexts. IEEE Access.

[83] Agrawal S., Chase M. (2017). FAME: Fast Attribute-Based Message Encryption. Cryptology ePrint Archive.

[84] Bethencourt J., Sahai A., Waters B. Ciphertext-policy attribute-based encryption. Proceedings of the Security and Privacy (SP’07) IEEE Symposium.

[85] Akinyele J.A., Garman C., Hohenberger S. (2005). Automating fast and secure translations from type-I to type-III pairing schemes. Proceedings of the 22nd ACM SIGSAC Conference on Computer and Communications Security.

[86] Zhang Y., Zheng D., Chen X., Li J., Li H. (2014). Computationally efficient ciphertext-policy attribute-based encryption with constant-size ciphertexts. Proceedings of the International Conference on Provable Security.

[87] Phuong T.V.X., Yang G., Susilo W., Chen X., Pernul G., Ryan P.Y.A., Weippl E. (2015). Attribute based broadcast encryption with short ciphertext and decryption key. Proceedings of the Computer Security–ESORICS 2015.

[88] Liu J.K., Yuen T.H., Zhang P., Liang K. (2018). Time-based direct revocable ciphertext-policy attribute-based encryption with short revocation list. Proceedings of the International Conference on Applied Cryptography and Network Security.

[89] Boldyreva A., Goyal V., Kumar V. Identity-based encryption with efficient revocation. Proceedings of the 2008 ACM Conference on Computer and Communications Security, CCS 2008.

[90] Attrapadung N., Imai H., Parker M.G. (2009). Attribute-based encryption supporting direct/indirect revocation modes. Proceedings of the Cryptography and Coding.

[91] Sahai A., Seyalioglu H., Waters B. (2012). Dynamic Credentials and Ciphertext Delegation for Attribute-Based Encryption. Cryptology ePrint Archive.

[92] Cui H., Deng R.H., Li Y., Qin B. (2016). Server-aided revocable attribute-based encryption. Proceedings of the Computer Security–ESORICS 2016–21st European Symposium on Research in Computer Security.

[93] Xu S., Yang G., Mu Y. (2019). Revocable attribute-based encryption with decryption key exposure resistance and ciphertext delegation. Inf. Sci..

[94] Cheng L., Meng F. (2021). Server-aided revocable attribute-based encryption revised: Multi-user setting and fully secure. Proceedings of the European Symposium on Research in Computer Security.

[95] Li J., Yao W., Han J., Zhang Y., Shen J. (2017). User collusion avoidance CP-ABE with efficient attribute revocation for cloud storage. IEEE Syst. J..

[96] Zhang Y., Chen X., Li J., Li H., Li F. (2014). Attribute-based data sharing with flexible and direct revocation in cloud computing. KSII Trans. Internet Inf. Syst..

[97] Phuong T.V.X., Yang G., Susilo W. Poster: Efficient ciphertext policy attribute-based encryption under decisional linear assumption. Proceedings of the 2014 ACM SIGSAC Conference on Computer and Communications Security.

[98] Hur J., Noh D.K. (2011). Attribute-based access control with efficient revocation in data outsourcing systems. IEEE Trans. Parallel Distrib. Syst..

[99] Zhou Z., Huang D. (2010). On efficient ciphertext-policy attribute-based encryption and broadcast encryption. Cryptology ePrint Archive.

[100] Qin B., Zhao Q., Zheng D., Cui H. (2017). Server-aided revocable attribute-based encryption resilient to decryption key exposure. Proceedings of the International Conference on Cryptology and Network Security.

[101] Parag V., Tiwari R., Hong W.C. (2023). Secure Authentication in IoT Based Healthcare Management Environment Using Integrated Fog Computing Enabled Blockchain System. Image Based Computing for Food and Health Analytics: Requirements, Challenges, Solutions and Practices: IBCFHA.

[102] Kumar A., Kumar S.A., Dutt V., Kumar Dubey A., Narang S. (2021). A Hybrid Secure Cloud Platform Maintenance Based on Improved Attribute-Based Encryption Strategies. https://reunir.unir.net/bitstream/handle/123456789/14366/ip2021_11_004.pdf?sequence=1.

